# Supplementary material for: The molecular basis, genetic control and pleiotropic effects of local gene co-expression
Source: Nat Commun. 2021 Aug 10;12:4842. doi: 10.1038/s41467-021-25129-x (PMC8355184; doi:10.1038/s41467-021-25129-x)
Supplement: Supplementary file 1 — Supplementary Information [file 41467_2021_25129_MOESM1_ESM.pdf]

# Supplementary Information

# The molecular basis, genetic control and pleiotropic effects of local gene co-expression

Ribeiro et al.

# Supplementary Figures

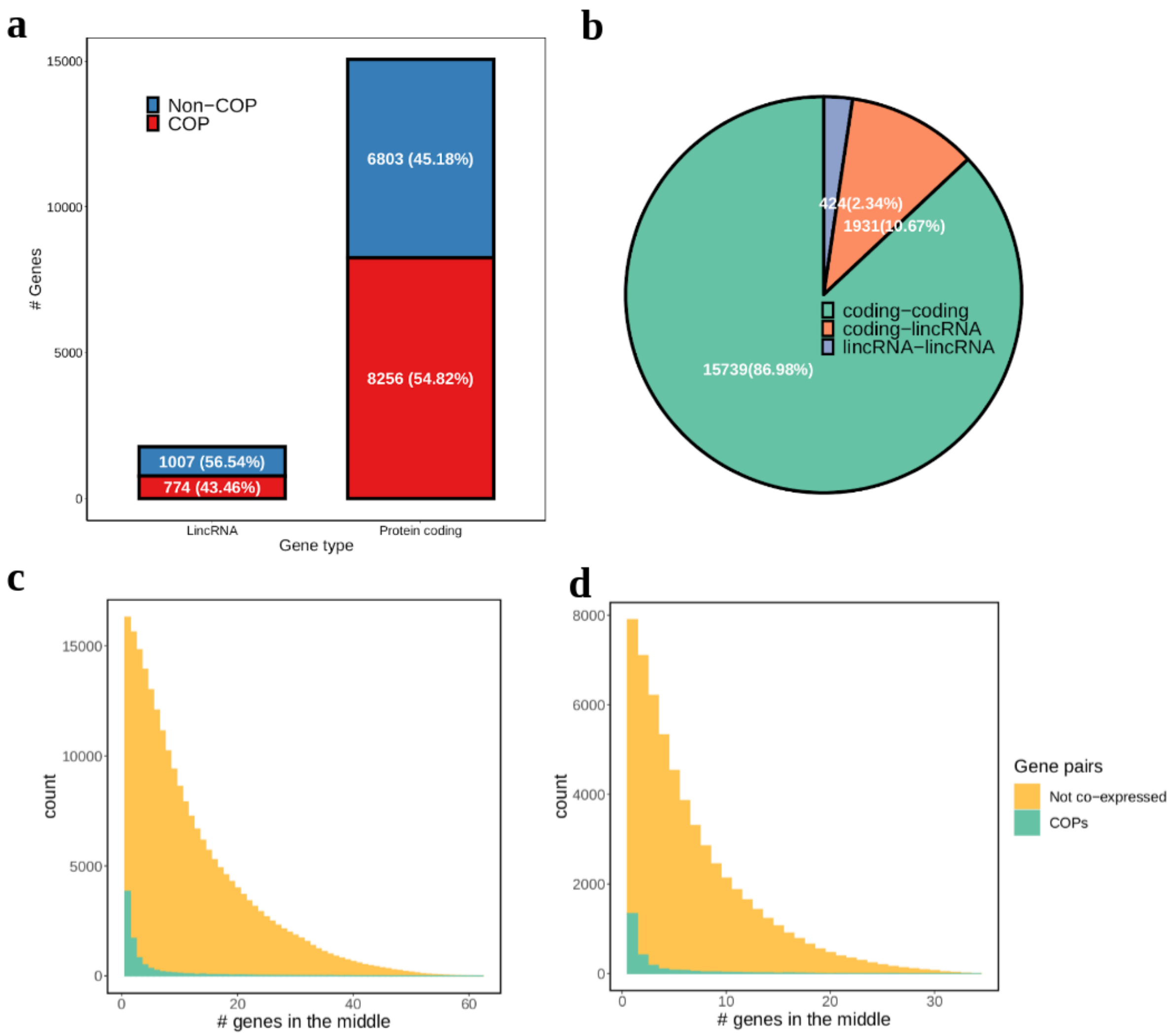


**Supplementary Fig. 1 Gene type and adjacency of COPs. a** numbers and proportions of co-expressed genes per gene type; **b** numbers and proportions of COPs per gene type. **c** distribution of the number of genes (TSSs of tested genes) found between gene pairs, considering all genes regardless of strand. 41% of Geuvadis COPs are formed between the nearest neighbours; **d** considering only positively stranded genes, as an example of considering gene neighbours only if being on the same strand. 53% are formed between the nearest neighbours.

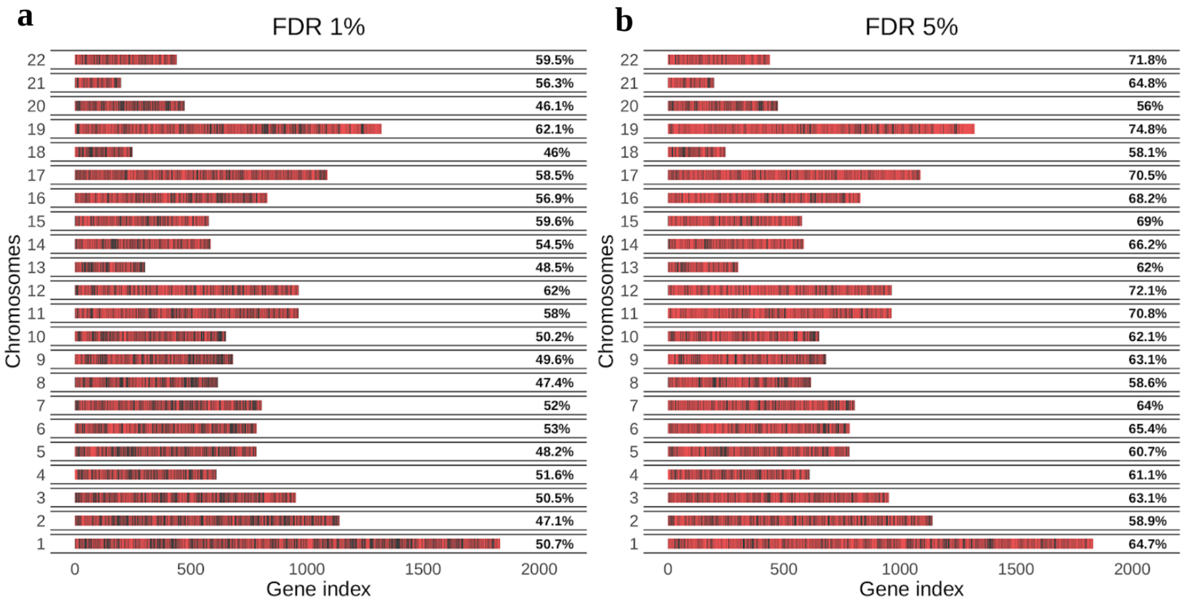


**Supplementary Fig. 2 Co-expressed gene percentage and distribution across chromosomes. a** COPs detected at 1% FDR; **b** COPs detected at 5% FDR. Red color represents a co-expressed gene, black a non-co-expressed gene. Values on the right side of each plot denote the percentage of genes that are co-expressed per chromosome.

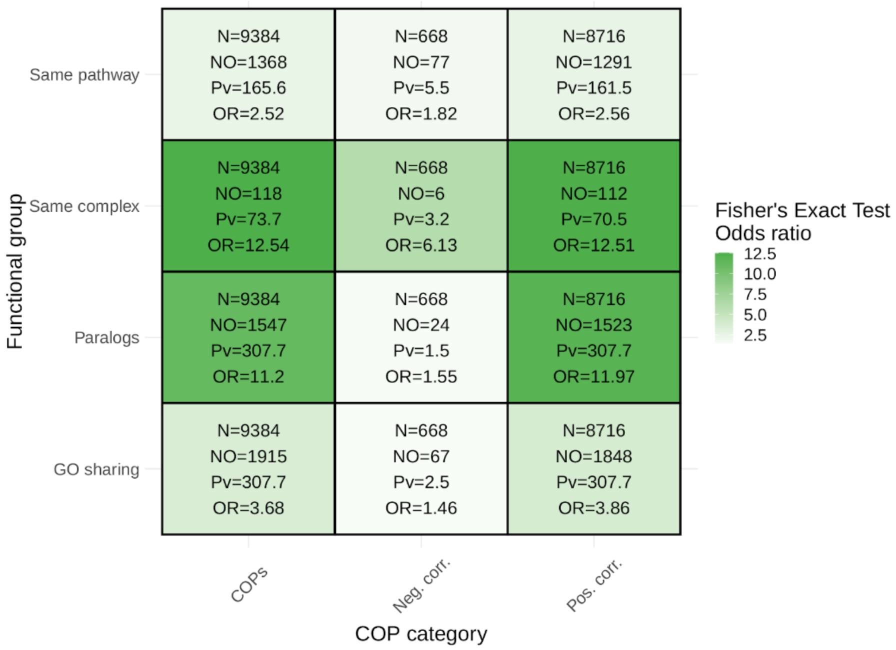


**Supplementary Fig. 3 Enrichment of COPs in functionally and evolutionarily-related datasets.** 'COPs' represent all COPs together, 'Neg. corr.' represents negatively correlated COPs only whereas 'Pos. corr.' represents positively correlated COPs. 'Same pathway' refers to enrichments for KEGG and Reactome pathways, 'Same complex' refers to enrichments for CORUM and Hu.MAP protein complexes, 'GO sharing' to enrichment of sharing of the same Biological Processes GO term. 'Paralogs' refers to enrichment in paralog genes (Methods). N, total number of COPs tested; NO, number of COPs overlapping with the functional group; Pv, One-sided Fisher's Exact test p-value (-log10 scale); OR, Fisher's Exact test odds ratio.

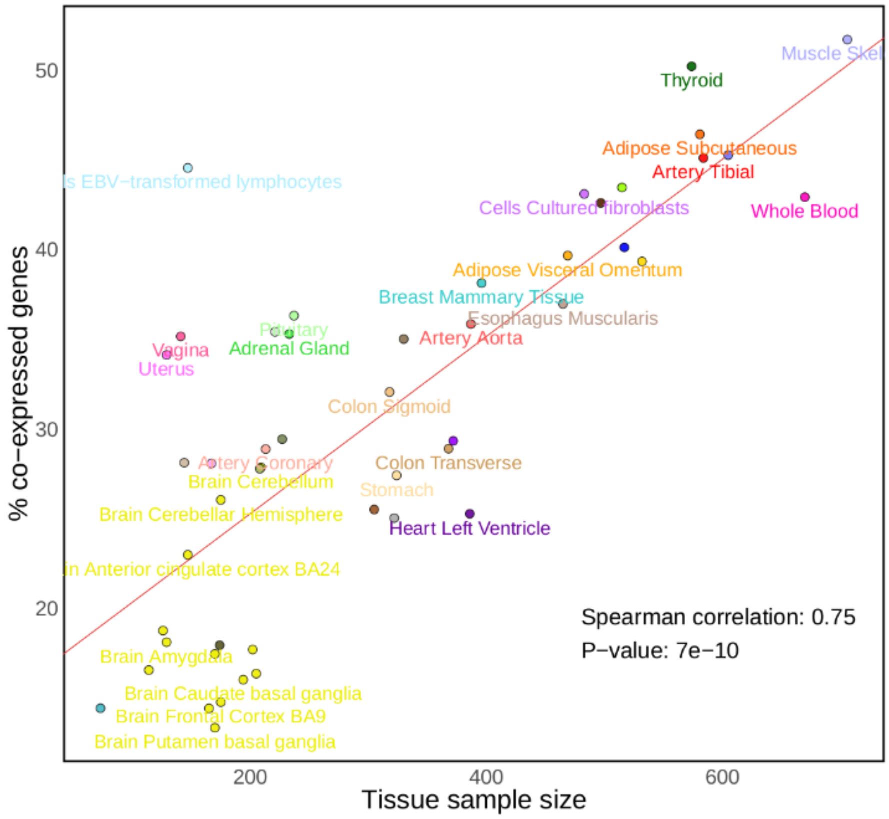


**Supplementary Fig. 4 Percentage of co-expressed genes per GTEx tissue sample size.**

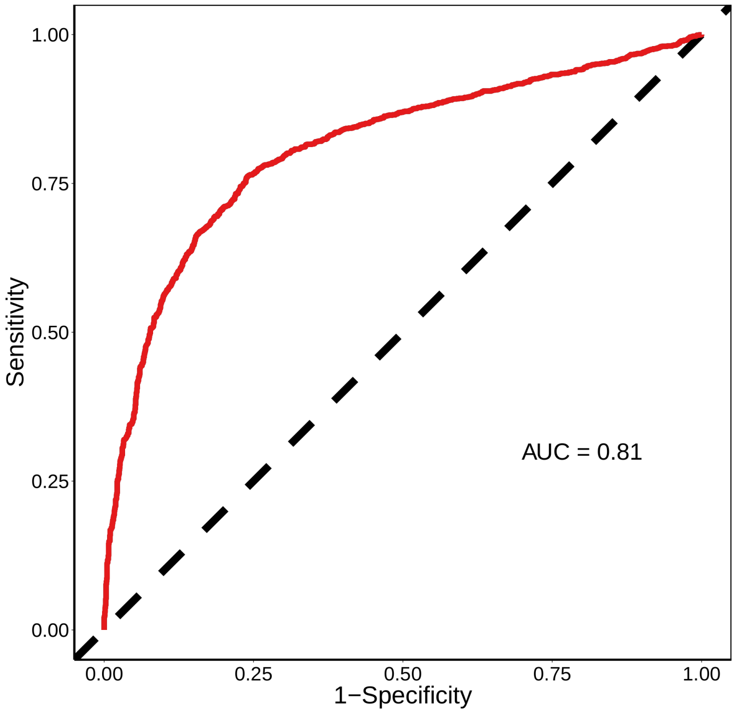


**Supplementary Fig. 5 Gene pair TSS absolute distance ROC curve for Geuvadis LCL COPs.** COP dataset before applying paralog and positive correlation filters, 9384 COPs (positives) and 9384 randomly sampled non-COPs (negatives).

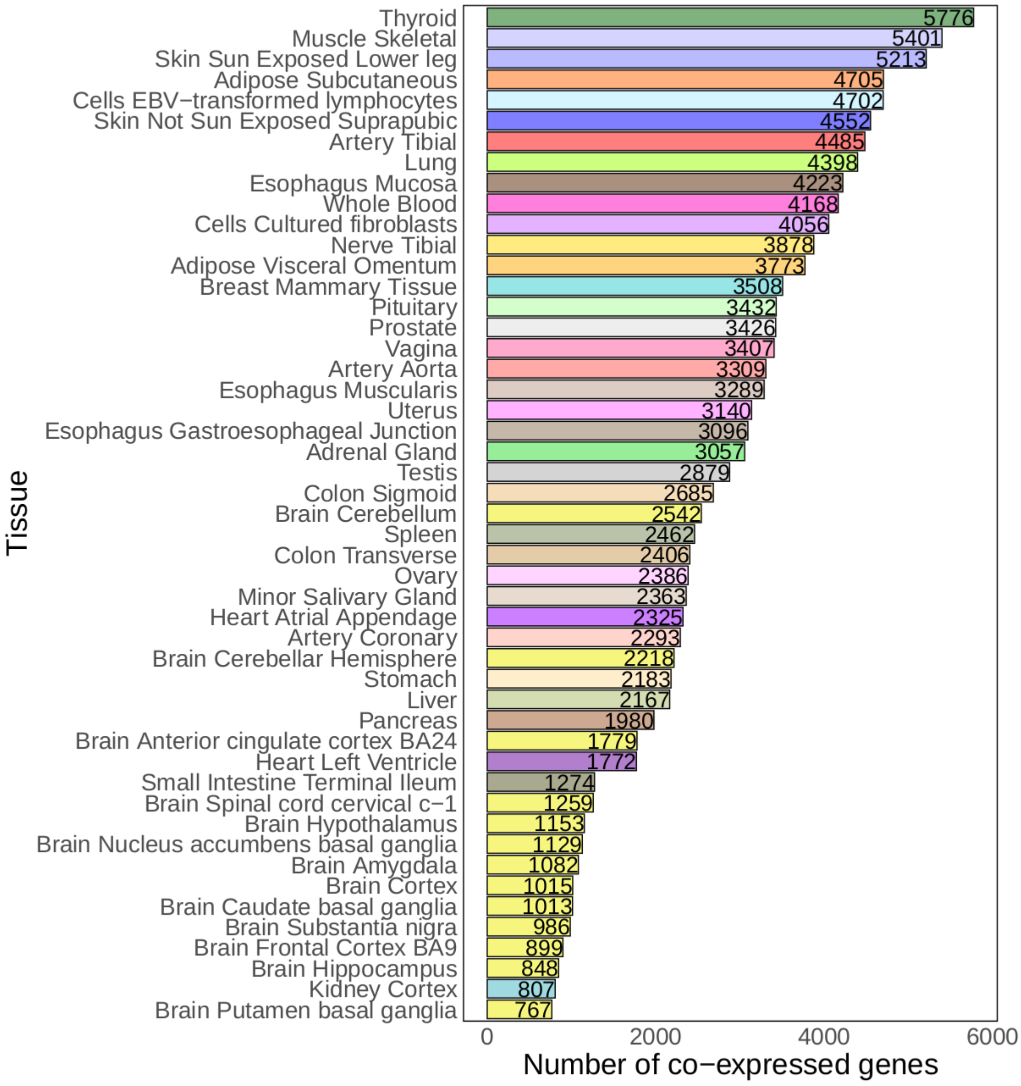


**Supplementary Fig. 6 Number of COPs identified in each GTEx tissue after paralog and positive correlation filters.**

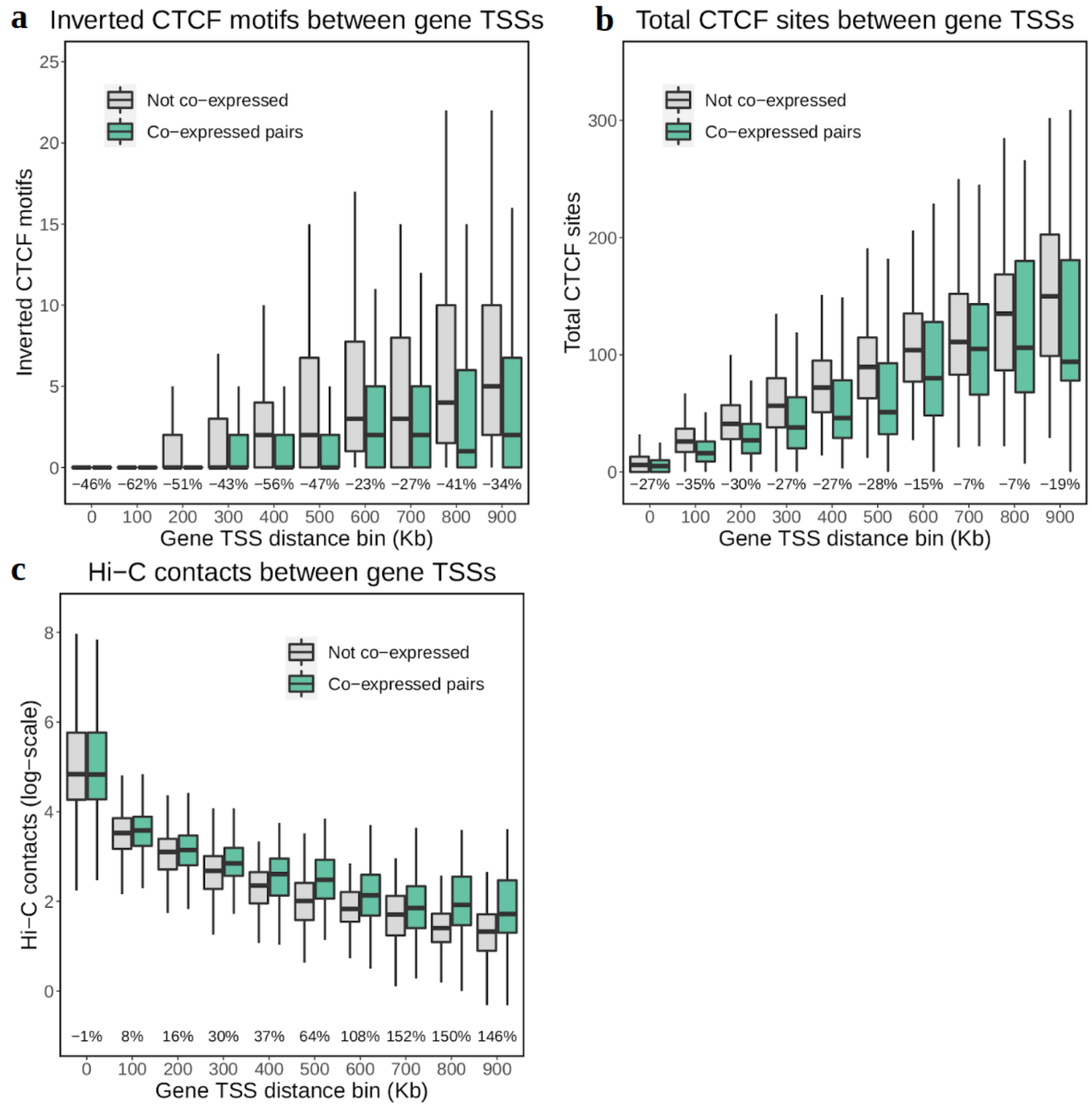


**Supplementary Fig. 7 Chromatin structure features (CTCF and Hi-C) on COPs and non-COPs across distance bins. a** inverted CTCF motifs (number of pairs of plus- and negatively-stranded motifs) between the two genes in the pair from MotifMap (predictions based on motif matching). N = 6668 for COPs and for non-COPs**; b** total CTCF sites between the two genes in the pair from ReMap, regardless of strand (LCL-specific experimental data; Methods). N = 6668 for COPs and for non-COPs; **c** Hi-C contact intensities between TSS regions of the two genes in the pair (log-scale; 5kb resolution) from Rao *et al.* 2014. Percentages at the bottom refer to the difference in means between COPs and non-COPs. N = 6668 for COPs and for non-COPs. For each boxplot, the length of the box corresponds to the interquartile range (IQR) with the centre line corresponding to the median, the upper and lower whiskers represent the largest value no further than 1.5 * IQR from the first and third quartile, respectively.

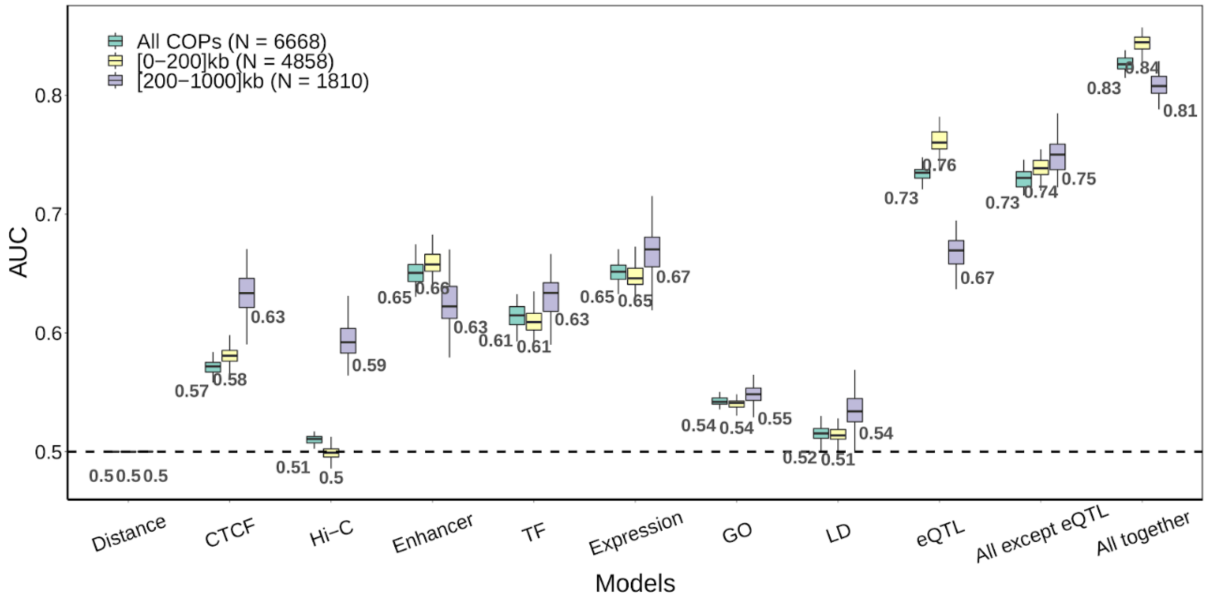


**Supplementary Fig. 8 Boxplots of the AUC values obtained for each molecular feature separated by two distance bins for Geuvadis LCLs**. Values below the boxplot represent the mean over the 50 randomisations. For each boxplot, the length of the box corresponds to the IQR with the centre line corresponding to the median, the upper and lower whiskers represent the largest value no further than 1.5 * IQR from the first and third quartile, respectively.

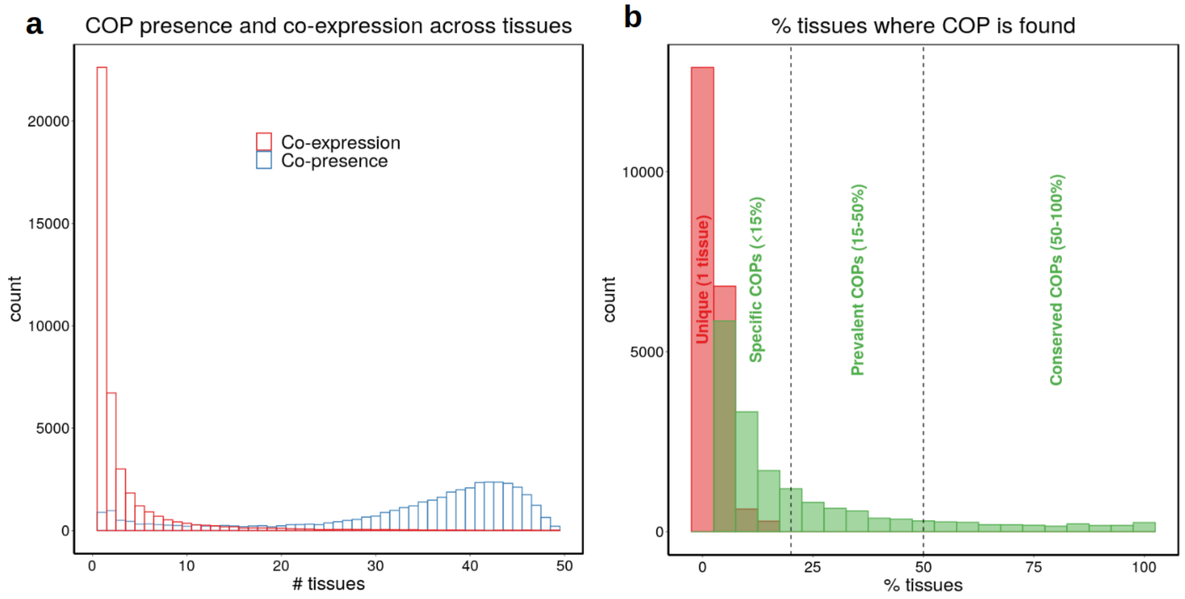


**Supplementary Fig. 9 Molecular feature results separating COPs into four categories based on their tissue prevalence across 49 tissues**. **a** in red, COP tissue frequency of all 40999 COPs is shown. In blue, the presence of these COPs across tissues is shown (i.e. based on gene expression of both genes, rather than significant co-expression); **b** distribution of the percentage of tissues where COPs are found (against the number of tissues where COPs are present), only COPs where both genes are present in at least 5 tissues were considered. This allowed the separation of COPs into the following categories: *i)* 'unique COPs', found in only one tissue (N = 20,781 across tissues), *ii)* 'specific COPs', found in more than 1 tissue but at most 15% tissues where both genes in the pair are present (range: 2-7 tissues, N = 10,111), *iii)* 'prevalent COPs', found in more than 1 tissue and between 15-50% tissues (N = 4,863) and *iv)* 'conserved COPs', found in more than 50% tissues (N = 2,441).

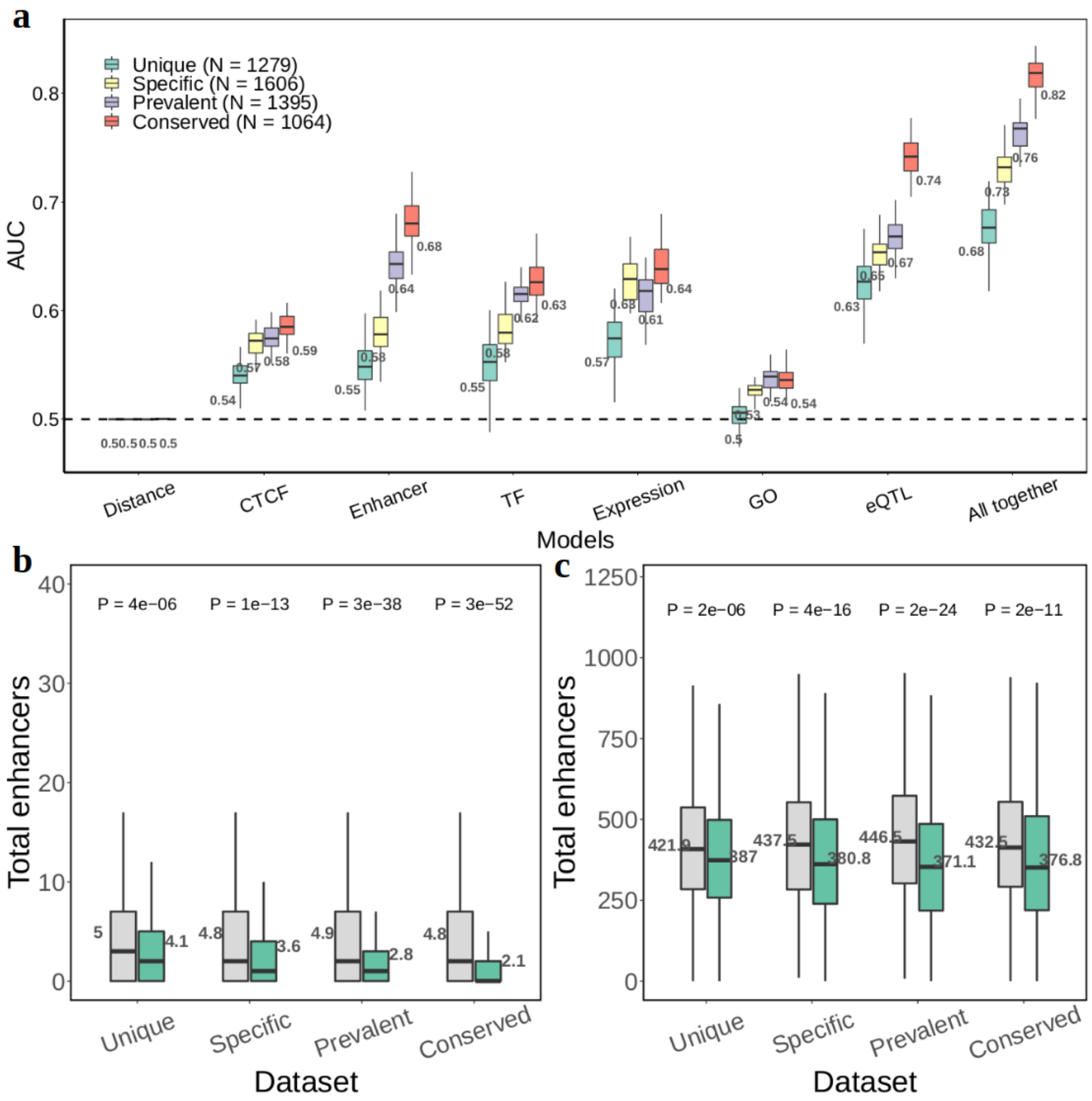


**Supplementary Fig. 10 Molecular feature results separating COPs into four categories based on their tissue prevalence across 49 tissues for Muscle Skeletal. a** boxplots of the AUC values obtained for each molecular feature on Muscle Skeletal COPs, separated by tissue prevalence categories. Values below the boxplot represent the mean over the 50 randomisations. Sample size of each category (number of positive and distance-matched negative) is found on the left corner of the plot; **b,c** boxplots of two molecular features of Muscle Skeletal COPs (green) and non-COPs (grey): total enhancers and total TFBS. Unique N = 1279, Specific N = 1606, Prevalent N = 1395, Conserved N = 1064, for both COPs and distance-matched non-COPs. Values next to the boxplots represent the mean. P-values were obtained from two-tailed Wilcoxon signed-rank tests. For each boxplot, the length of the box corresponds to the IQR with the centre line corresponding to the median, the upper and lower whiskers represent the largest or lowest value no further than 1.5 * IQR from the third and first quartile, respectively.

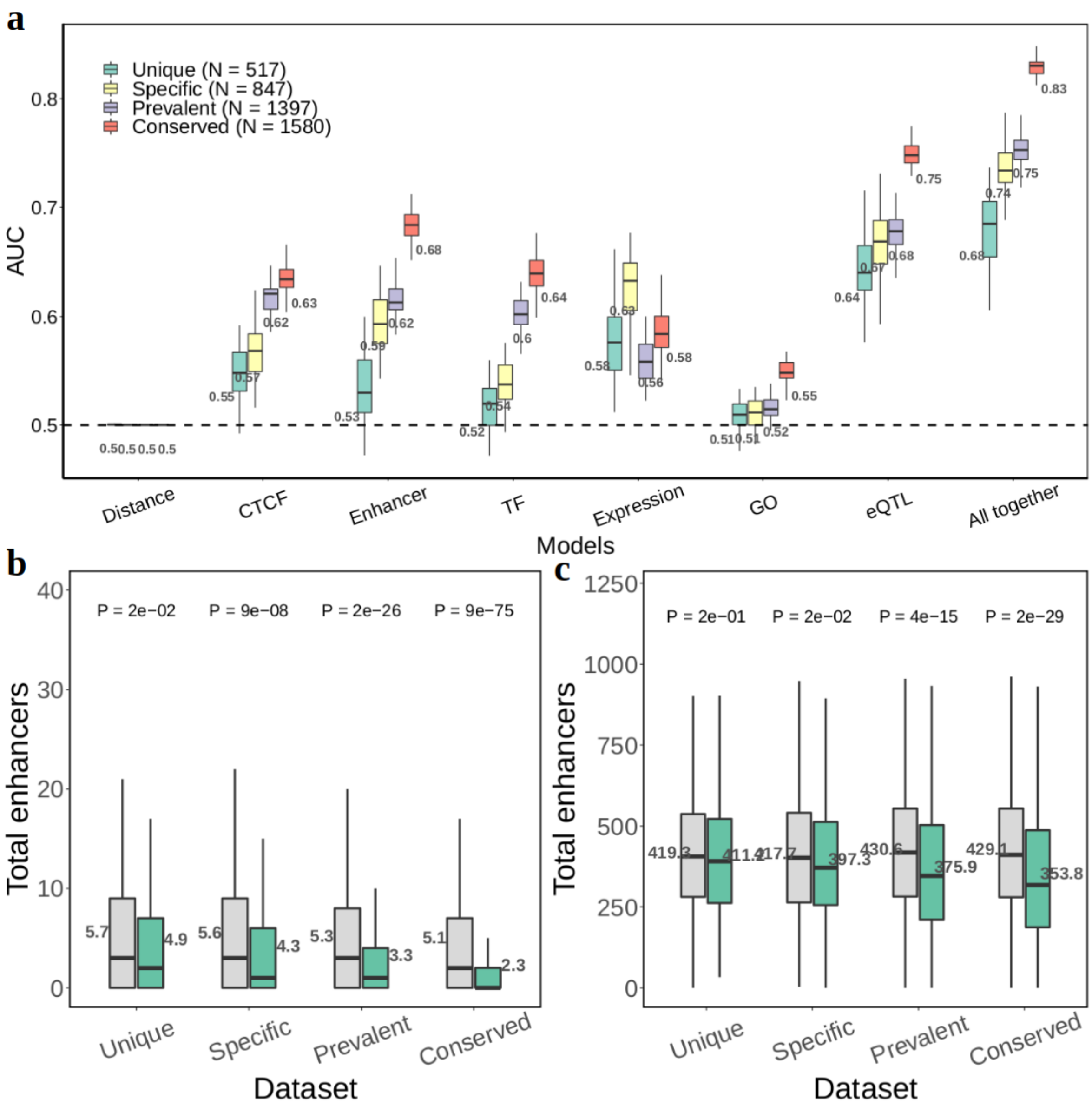


**Supplementary Fig. 11 Molecular feature results separating COPs into four categories based on their tissue prevalence across 49 tissues for Lung**. **a** boxplots of the AUC values obtained for each molecular feature on Lung COPs, separated by tissue prevalence categories. Values below the boxplot represent the mean over the 50 randomisations. Sample size of each category (number of positive and distance-matched negative) is found on the left corner of the plot; **b,c** boxplots of two molecular features of Lung COPs (green) and non-COPs (grey): total enhancers and total TFBS. Unique N = 1279, Specific N = 1606, Prevalent N = 1395, Conserved N = 1064, for both COPs and distance-matched non-COPs. Values next to the boxplots represent the mean. P-values were obtained from two-tailed Wilcoxon signed-rank tests. For each boxplot, the length of the box corresponds to the IQR with the centre line corresponding to the median, the upper and lower whiskers represent the largest or lowest value no further than 1.5 * IQR from the third and first quartile, respectively.

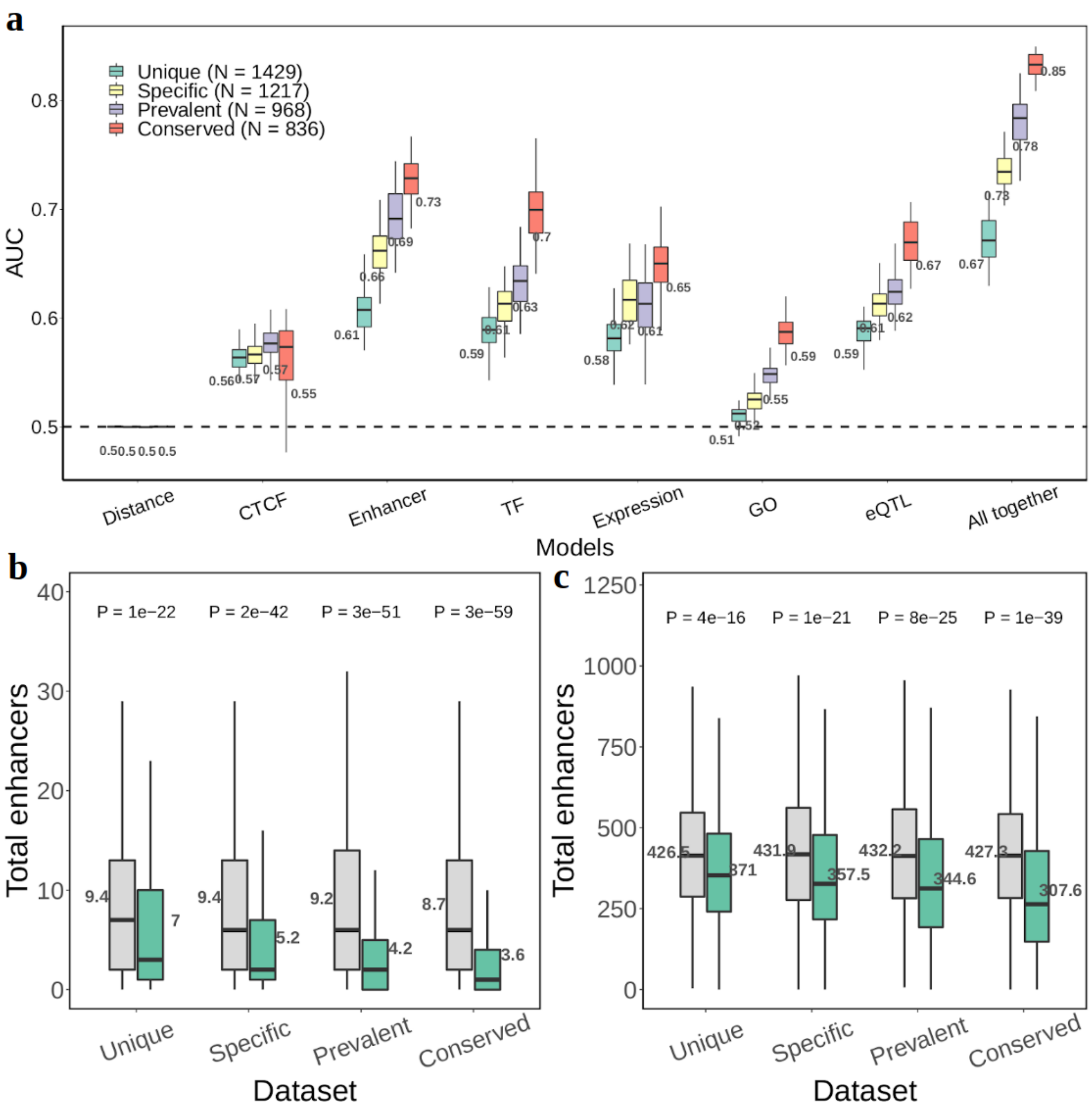


**Supplementary Fig. 12 Molecular feature results separating COPs into four categories based on their tissue prevalence across 49 tissues for GTEx LCLs.** **a** boxplots of the AUC values obtained for each molecular feature on LCL COPs, separated by tissue prevalence categories. Values below the boxplot represent the mean over the 50 randomisations. Sample size of each category (number of positive and distance-matched negative) is found on the left corner of the plot; **b,c** boxplots of two molecular features of GTEx LCL COPs (green) and non-COPs (grey): total enhancers and total TFBS. Unique N = 1279, Specific N = 1606, Prevalent N = 1395, Conserved N = 1064, for both COPs and distance-matched non-COPs. Values next to the boxplots represent the mean. P-values were obtained from two-tailed Wilcoxon signed-rank tests. For each boxplot, the length of the box corresponds to the IQR with the centre line corresponding to the median, the upper and lower whiskers represent the largest or lowest value no further than 1.5 * IQR from the third and first quartile, respectively.

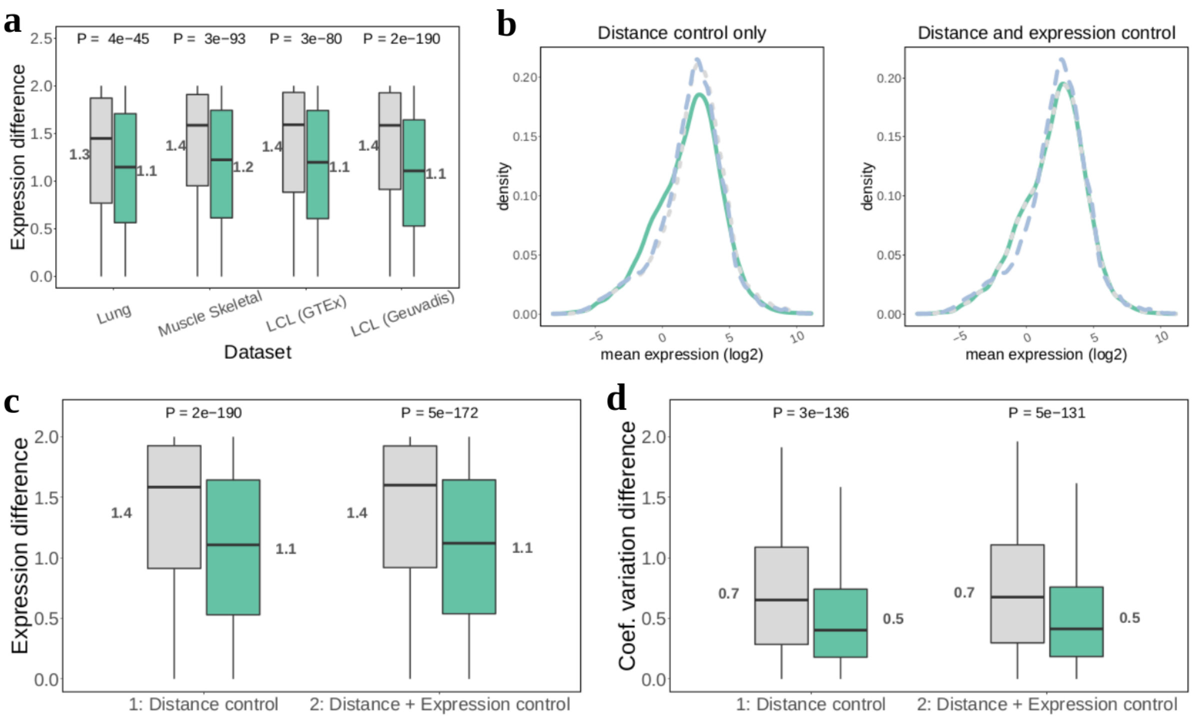


**Supplementary Fig. 13 Expression level difference and coefficient of variation difference between COPs and non-COPs**. **a** boxplots of the expression level difference between the two genes in the pair, for COPs (green) and non-COPs (grey) across 4 datasets. Expression level difference was calculated as the absolute difference between the average expression level of the genes in the pair, divided by the average expression level of the pair. In all cases, values next to the b oxplots represent means. Sample sizes: Lung = 4398, Muscle Skeletal = 5401, LCL (GTEx) = 4702, LCL (Geuvadis) = 6668; **b** details of the mean expression level control (between the two genes in the pair) used for this specific analysis. After controlling for both distance (at most 5% difference in distance allowed between COP and matching non-COP) and mean expression level (at most 10% difference allowed), the mean expression distribution between COPs (green) and non-COPs (grey) clearly matches (right plot). In the step of picking non-COPs matched for both distance and expression level, 545 COPs were lost. For a comparison, the mean expression distribution for all other gene pairs (N = 183748) is shown in blue; **c** boxplots of the expression level difference between the two genes in the pair for Geuvadis LCLs COPs and non-COPs, before (N = 6668) and after (N = 6123) controlling for mean expression level (Methods); **d** boxplots of the coefficient of variation difference between the two genes in the pair for Geuvadis LCLs COPs (N = 6668) and non-COPs, before (N = 6668) and after (N = 6123) controlling for mean expression level. The difference between COP and non-COP is highly significant in all cases two-sided Wilcoxon signed-rank tests p-values $<2.2e^{-16}$. Mean RPKMs were used for Geuvadis LCLs and median TPM were used for GTEx tissues. For each boxplot, the length of the box corresponds to the IQR with the centre line corresponding to the median, the upper and lower whiskers represent the largest or lowest value no further than 1.5 * IQR from the third and first quartile, respectively.

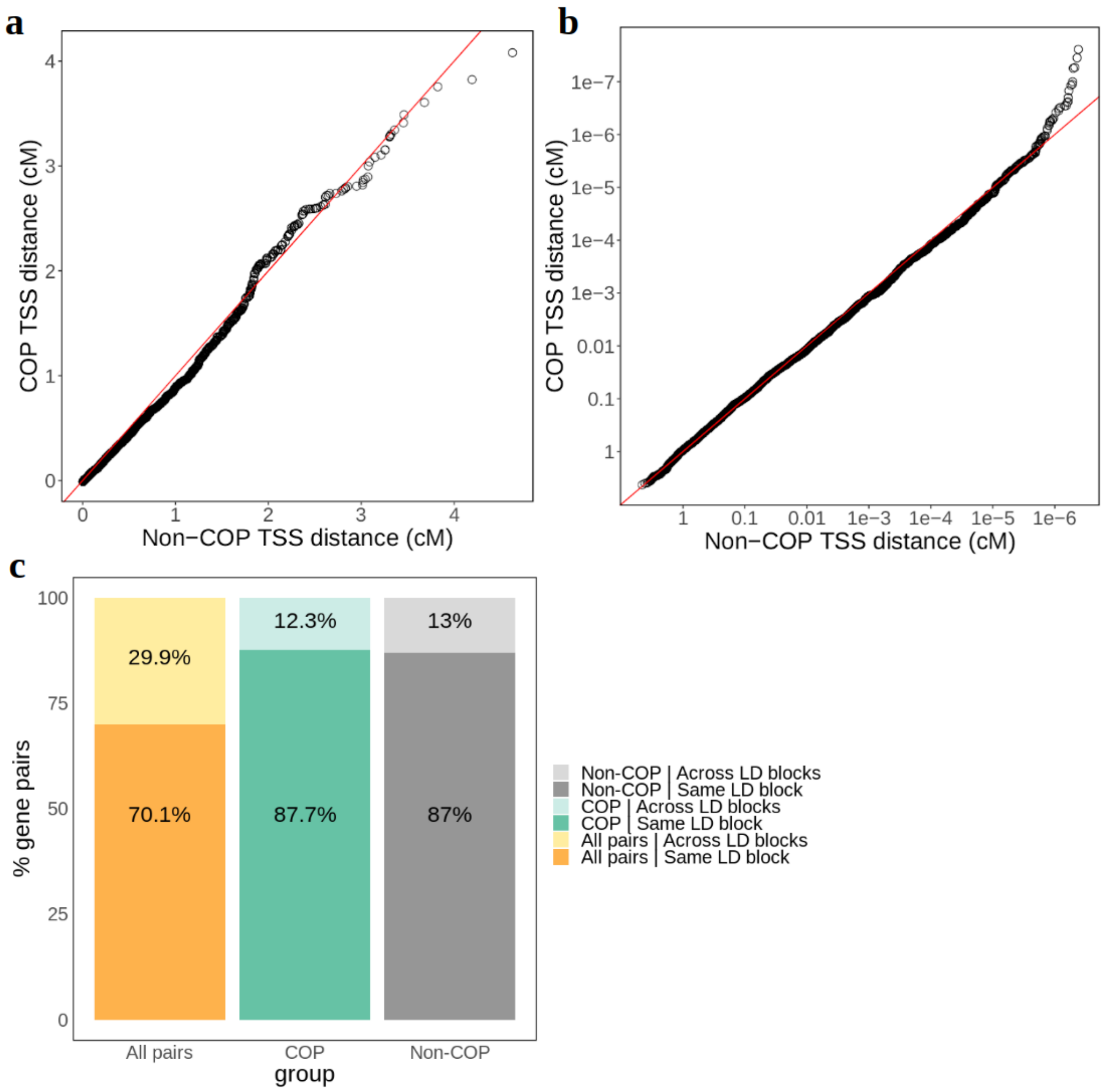


**Supplementary Fig. 14 Comparison of centimorgan (cM) distance between TSSs of Geuvadis LCL COPs and non-COPs. a** absolute distance in normal scale; **b** absolute distance in -log10 scale. In both cases 6668 COPs and 6668 distance matched non-COPs were used and linear regression slope = 1.02; **c** number of gene pairs found in the same LD block or across LD blocks (based on Berisa & Pickrell 2016, Methods). For a comparison, the number for all other gene pairs (N = 183748) is shown in yellow.

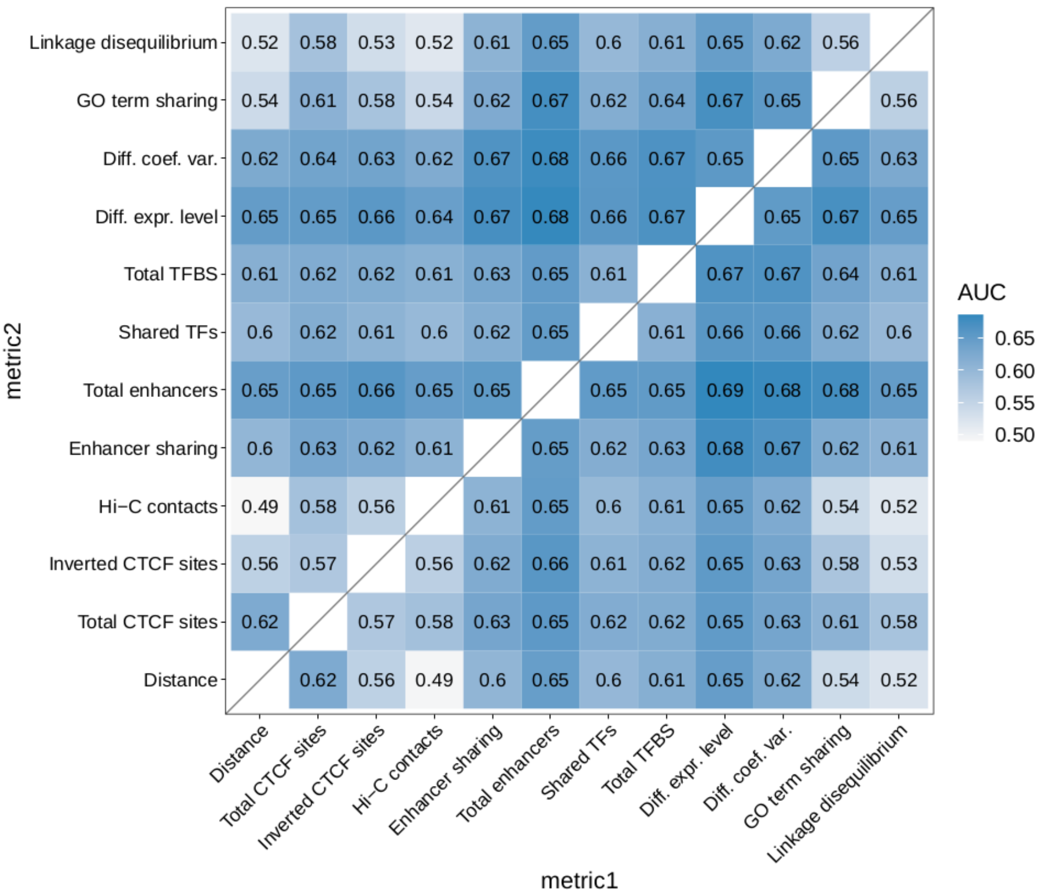


**Supplementary Fig. 15 Mean AUC of pairwise combinations of molecular feature metrics in Geuvadis LCLs**. AUCs are averages of 50 training-test set randomisations. The upper and lower triangles come from two separate sets of randomisations.

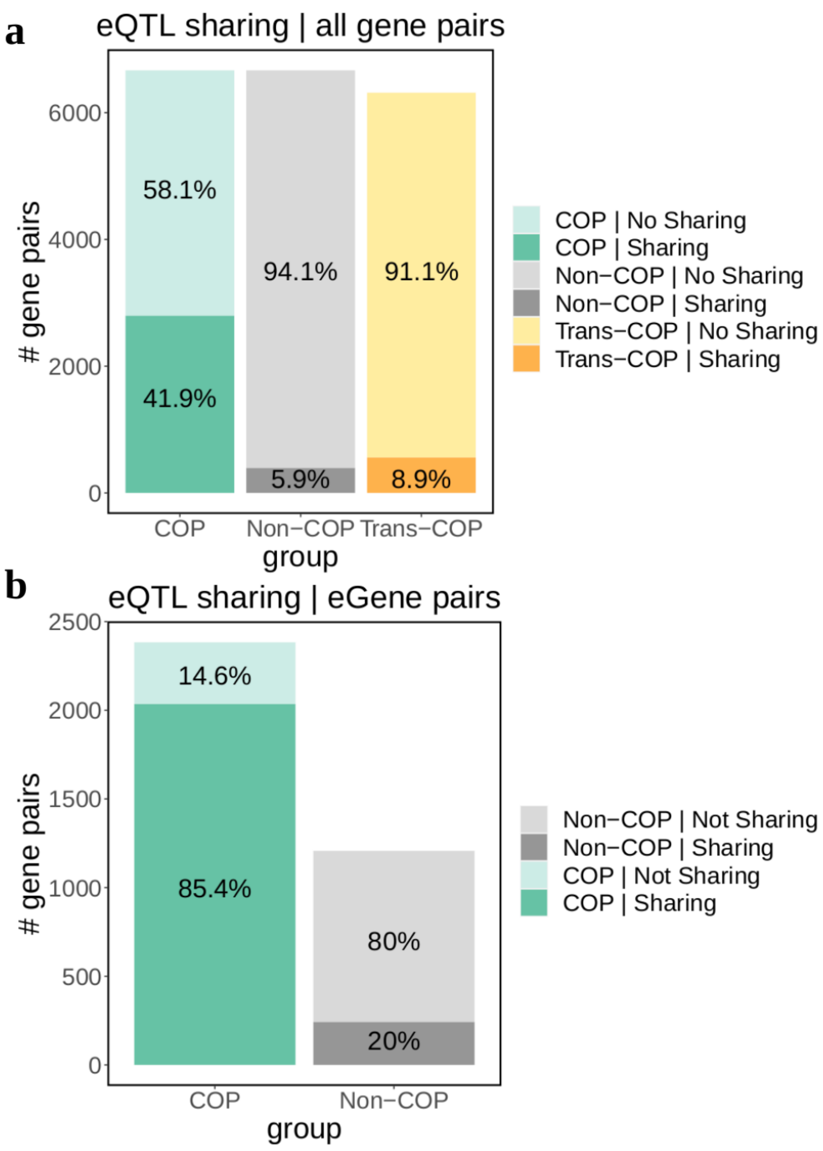


**Supplementary Fig. 16 eQTL sharing in COP, non-COPs and trans-COPs. a** number and percentages of Geuvadis LCLs COPs (N = 6668), distance-matched non-COPs (N = 6668) and correlation-matched trans-COPs (N = 6316) in eQTL sharing. Only 6316 trans-COPs could be matched to cis-COPs by correlation (maximum difference of 5% correlation value between cis-COPs and trans-COPs; Methods); **b** eQTL sharing in Geuvadis LCL COPs and non-COPs when only considering cases where both genes are eGenes.

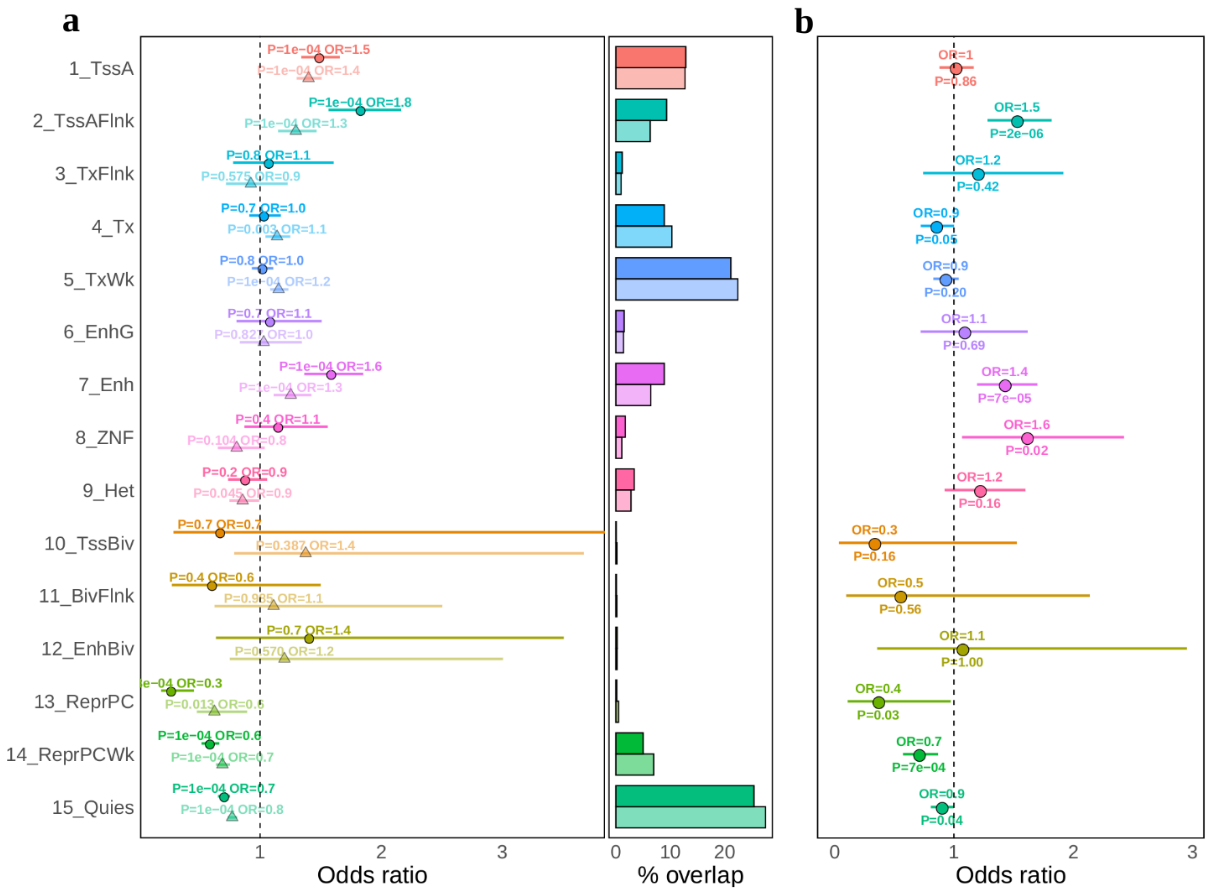


**Supplementary Fig. 17 Functional enrichments of Geuvadis LCL shared eQTLs on Roadmap epigenomics annotations. a** overlap enrichment of shared lead eQTLs (solid color, round points) and other lead eQTLs (pale color, triangles) in 15-state Roadmap epigenomics functional annotations for Geuvadis LCL (Methods). Odd ratios are calculated based on the observed versus expected overlap (10000 QTLtools fenrich permutations; Methods) between eQTLs and each functional annotation, through two-sided Fisher's exact tests (no multiple test adjustment). Error bars are 95% confidence intervals. The right part of the plot denotes the percentage of overlap between eQTLs and each functional annotation; **b** Two-sided Fisher's exact test odds ratio and p-value for the enrichment of shared lead eQTLs in each functional annotation, compared to other lead eQTLs, for Geuvadis LCL. Error bars of odds ratio are 95% confidence intervals. Annotation legend: 1_TssA: Active TSS, 2_TssAFlnk: Flanking Active TSS, 3_TxFlnk: Transcr. at gene 5' and 3', 4_Tx: Strong transcription, 5_TxWk: Weak transcription, 6_EnhG: Genic enhancers, 7_Enh: Enhancers, 8_ZNF/Rpts: ZNF genes & repeats, 9_Het: Heterochromatin, 10_TssBiv: Bivalent/Poised TSS, 11_BivFlnk: Flanking Bivalent TSS/Enh, 12_EnhBiv: Bivalent Enhancer, 13_ReprPC: Repressed PolyComb, 14_ReprPCWk: Weak Repressed PolyComb, 15_ Quies: Quiescent/Low.

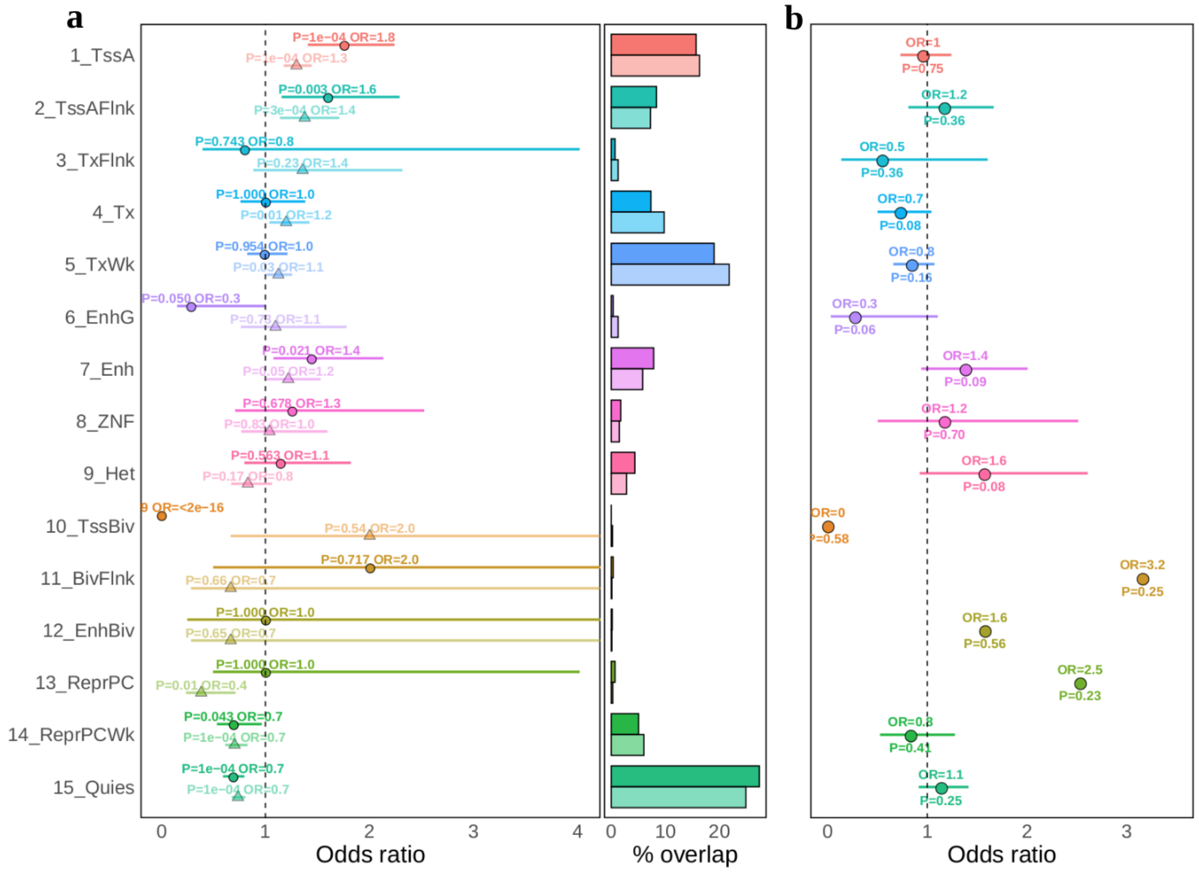


**Supplementary Fig. 18 Functional enrichments of GTEx LCL shared eQTLs on Roadmap epigenomics annotations. a** overlap enrichment of shared lead eQTLs (solid color, round points) and other lead eQTLs (pale color, triangles) in 15-state Roadmap epigenomics functional annotations for GTEx LCL (Methods). Odd ratios are calculated based on the observed versus expected overlap (10000 QTLtools fenrich permutations; Methods) between eQTLs and each functional annotation, through two-sided Fisher's exact tests (no multiple test adjustment). Error bars are 95% confidence intervals. The right part of the plot denotes the percentage of overlap between eQTLs and each functional annotation; **b** Two-sided Fisher's exact test odds ratio and p-value for the enrichment of shared lead eQTLs in each functional annotation, compared to other lead eQTLs, for GTEx LCL. Error bars of odds ratio are 95% confidence intervals. Annotation legend: 1_TssA: Active TSS, 2_TssAFlnk: Flanking Active TSS, 3_TxFlnk: Transcr. at gene 5' and 3', 4_Tx: Strong transcription, 5_TxWk: Weak transcription, 6_EnhG: Genic enhancers, 7_Enh: Enhancers, 8_ZNF/Rpts: ZNF genes & repeats, 9_Het: Heterochromatin, 10_TssBiv: Bivalent/Poised TSS, 11_BivFlnk: Flanking Bivalent TSS/Enh, 12_EnhBiv: Bivalent Enhancer, 13_ReprPC: Repressed PolyComb, 14_ReprPCWk: Weak Repressed PolyComb, 15_ Quies: Quiescent/Low.

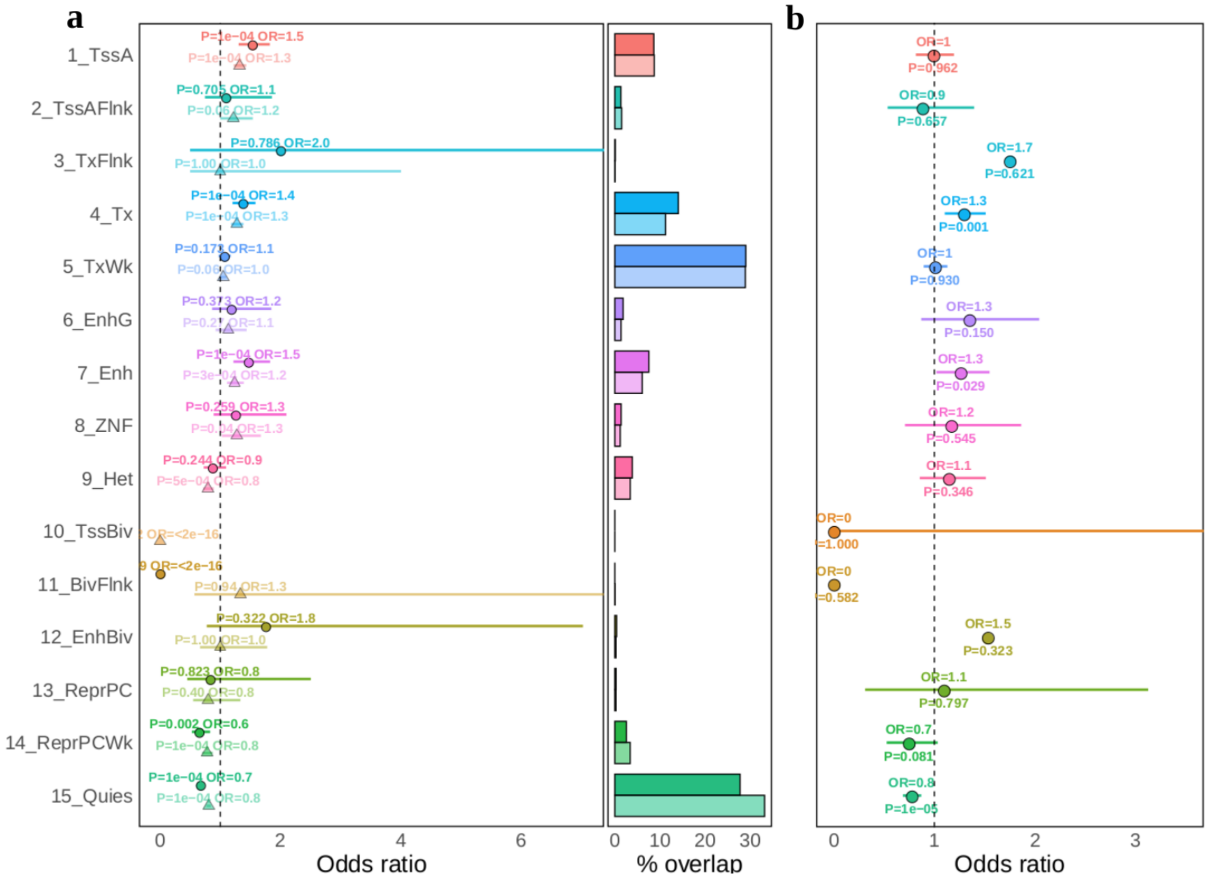


**Supplementary Fig. 19 Functional enrichments of Lung shared eQTLs on Roadmap epigenomics annotations. a** overlap enrichment of shared lead eQTLs (solid color, round points) and other lead eQTLs (pale color, triangles) in 15-state Roadmap epigenomics functional annotations for Lung (Methods). Odd ratios are calculated based on the observed versus expected overlap (10000 QTLtools fenrich permutations; Methods) between eQTLs and each functional annotation, through two-sided Fisher's exact tests (no multiple test adjustment). Error bars are 95% confidence intervals. The right part of the plot denotes the percentage of overlap between eQTLs and each functional annotation; **b** Two-sided Fisher's exact test odds ratio and p-value for the enrichment of shared lead eQTLs in each functional annotation, compared to other lead eQTLs, for Lung. Error bars of odds ratio are 95% confidence intervals. Annotation legend: 1_TssA: Active TSS, 2_TssAFlnk: Flanking Active TSS, 3_TxFlnk: Transcr. at gene 5' and 3', 4_Tx: Strong transcription, 5_TxWk: Weak transcription, 6_EnhG: Genic enhancers, 7_Enh: Enhancers, 8_ZNF/Rpts: ZNF genes & repeats, 9_Het: Heterochromatin, 10_TssBiv: Bivalent/Poised TSS, 11_BivFlnk: Flanking Bivalent TSS/Enh, 12_EnhBiv: Bivalent Enhancer, 13_ReprPC: Repressed PolyComb, 14_ReprPCWk: Weak Repressed PolyComb, 15_ Quies: Quiescent/Low.

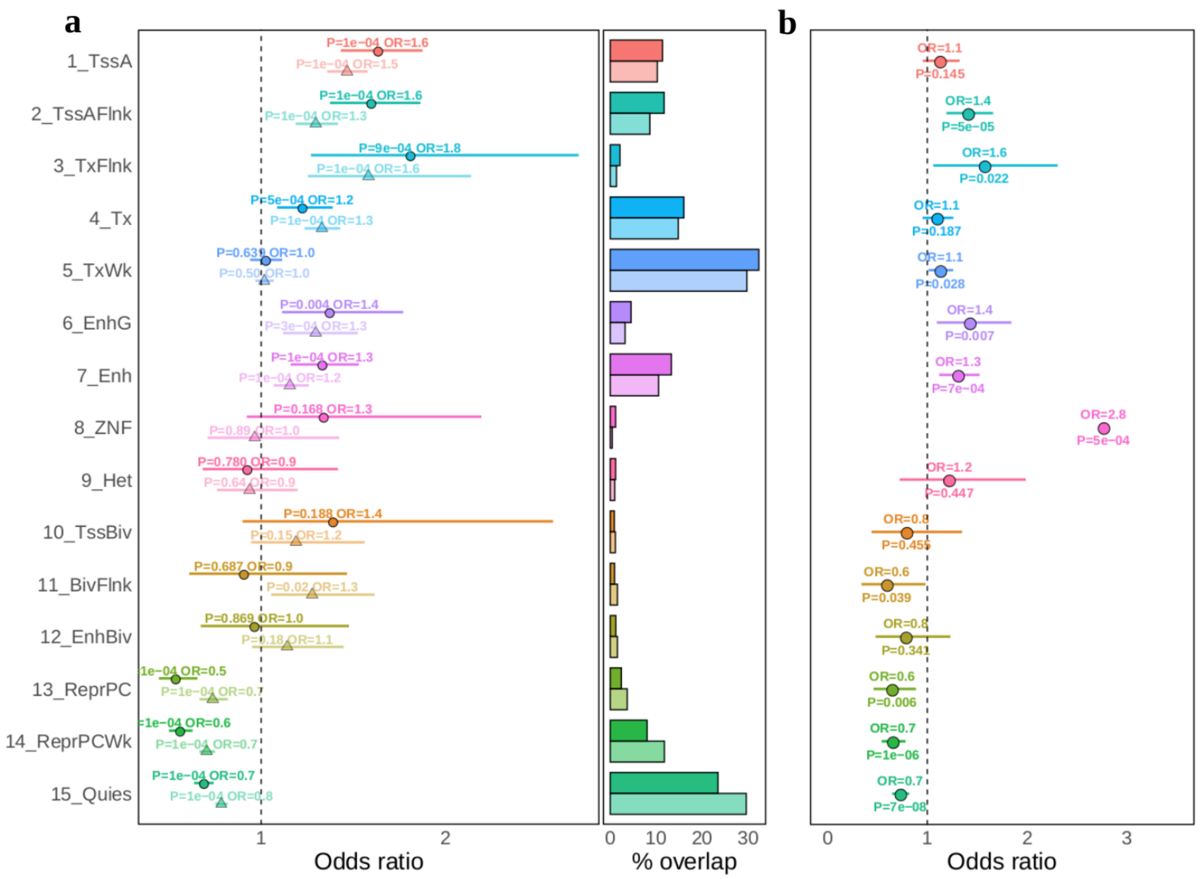


**Supplementary Fig. 20 Functional enrichments of Muscle Skeletal shared eQTLs on Roadmap epigenomics annotations. a** overlap enrichment of shared lead eQTLs (solid color, round points) and other lead eQTLs (pale color, triangles) in 15-state Roadmap epigenomics functional annotations for Muscle Skeletal (Methods). Odd ratios are calculated based on the observed versus expected overlap (10000 QTLtools fenrich permutations; Methods) between eQTLs and each functional annotation, through two-sided Fisher's exact tests (no multiple test adjustment). Error bars are 95% confidence intervals. The right part of the plot denotes the percentage of overlap between eQTLs and each functional annotation; **b** Two-sided Fisher's exact test odds ratio and p-value for the enrichment of shared lead eQTLs in each functional annotation, compared to other lead eQTLs, for Muscle Skeletal. Error bars of odds ratio are 95% confidence intervals. Annotation legend: 1_TssA: Active TSS, 2_TssAFlnk: Flanking Active TSS, 3_TxFlnk: Transcr. at gene 5' and 3', 4_Tx: Strong transcription, 5_TxWk: Weak transcription, 6_EnhG: Genic enhancers, 7_Enh: Enhancers, 8_ZNF/Rpts: ZNF genes & repeats, 9_Het: Heterochromatin, 10_TssBiv: Bivalent/Poised TSS, 11_BivFlnk: Flanking Bivalent TSS/Enh, 12_EnhBiv: Bivalent Enhancer, 13_ReprPC: Repressed PolyComb, 14_ReprPCWk: Weak Repressed PolyComb, 15_ Quies: Quiescent/Low.

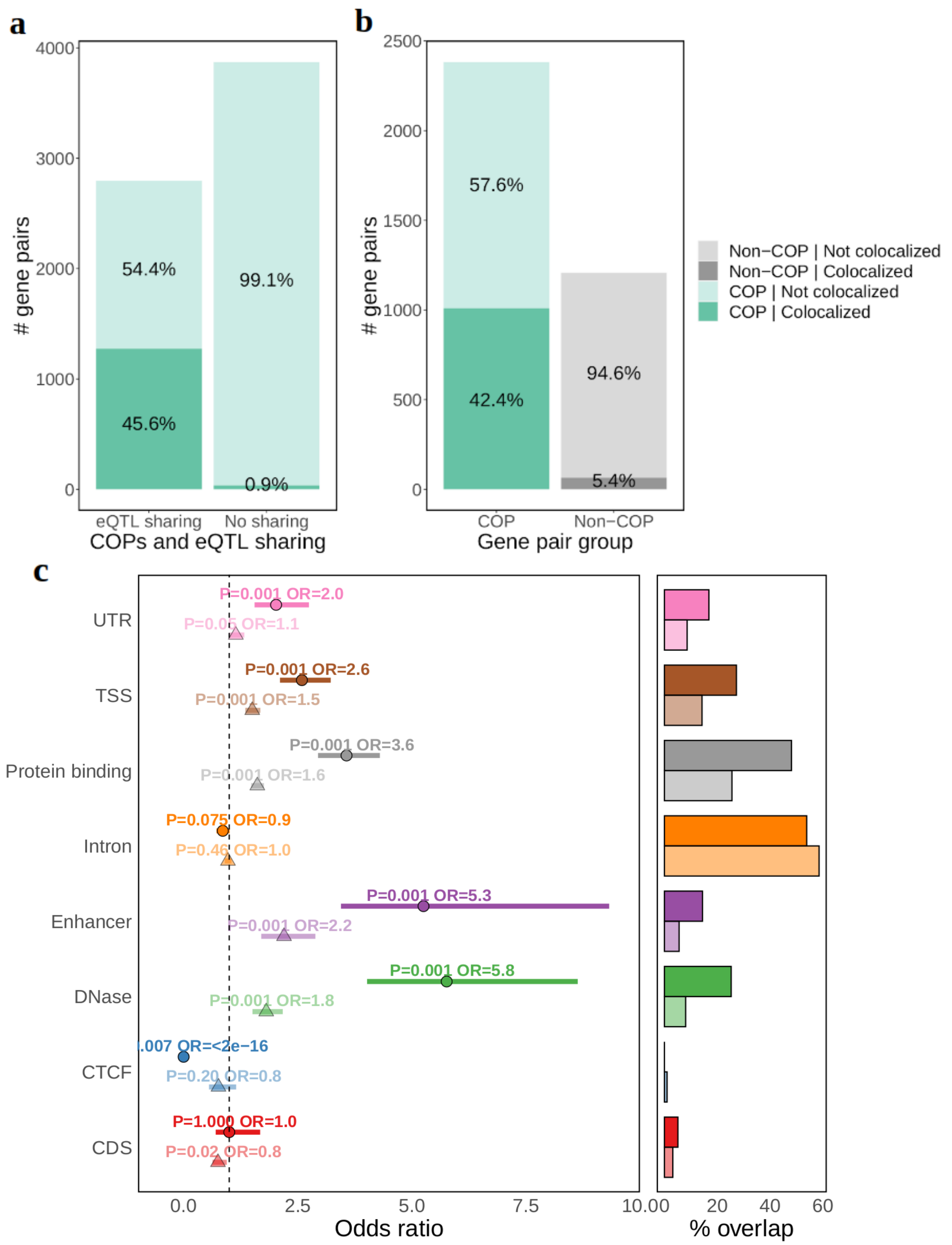


**Supplementary Fig. 21 Comparison of gene pair colocalization between eQTL sharing status, COPs and non-COPs and their functional enrichments.** **a** number of colocalized COPs (COLOC PP4 > 0.5) split by eQTL sharing status. Out of 6668 Gevaudis LCL COPs, 2796 are in eQTL sharing and 3871 are not; **b** numbers of colocalized COPs and non-COPs, only including gene pairs where both genes are eGenes, which is more likely for COPs. N = 2383 for COPs, N = 1207 for distance-matched non-COPs; **c** Geuvadis LCL functional enrichment for shared eQTLs with coloc PP4>0.5 (solid color, round points, N = 451) and shared eQTLs with coloc PP4<=0.5 (pale color, triangles, N = 2303). Odd ratios are calculated based on the observed versus expected overlap (10000 QTLtools fenrich permutations; Methods) between eQTLs and each functional annotation, through two-sided Fisher's exact tests (no multiple test adjustment). Error bars are 95% confidence intervals.

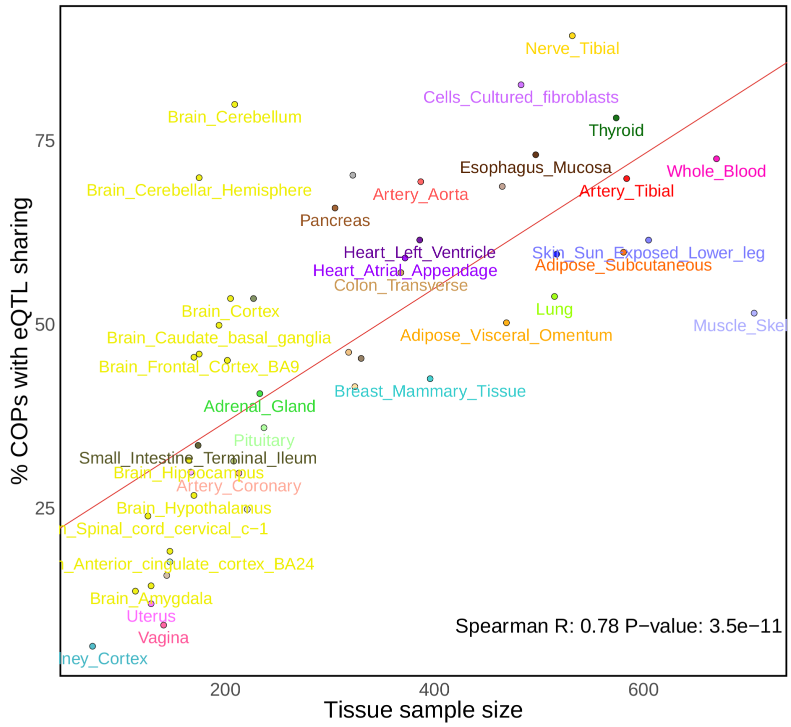


**Supplementary Fig. 22 Percentage of COPs with eQTL sharing per tissue sample size.** Correlation test p-value is two-sided.

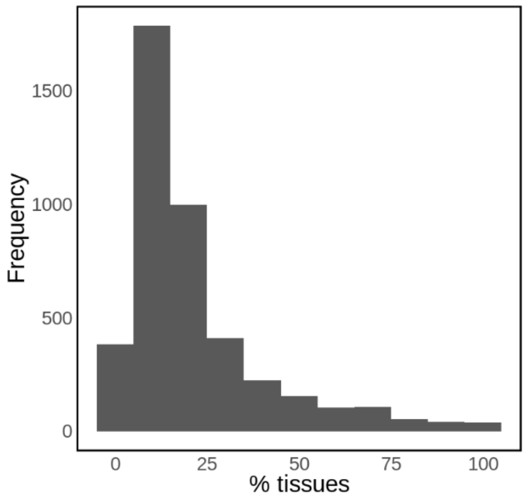


**Supplementary Fig. 23 Replication of shared lead eQTL-COPs across tissues**. Percentage of tissues where COP associates with the same shared lead eQTL, out of all tissues where the COP is present. Only COPs present in >5 tissues were considered (N = 4298), in order to exclude cases where for example the eQTL is associated in 100% tissues while in fact the COP only occurs in a few tissues. On average, a COP is associated with the same lead eQTL in 21.8% of the tissues. When several eQTLs are available for a COP, we consider only the most shared eQTL.

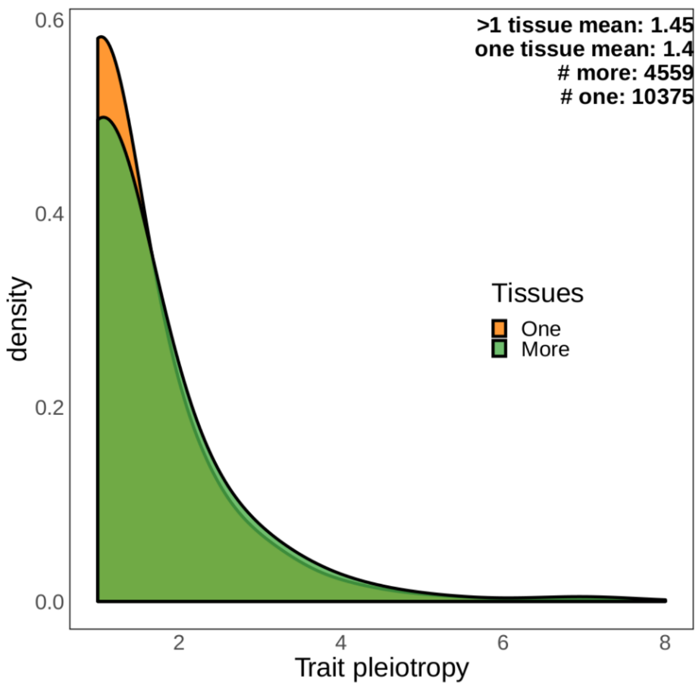


**Supplementary Fig. 24 Comparison of trait pleiotropy between lead eQTLs present in one tissue or more than one tissue.**

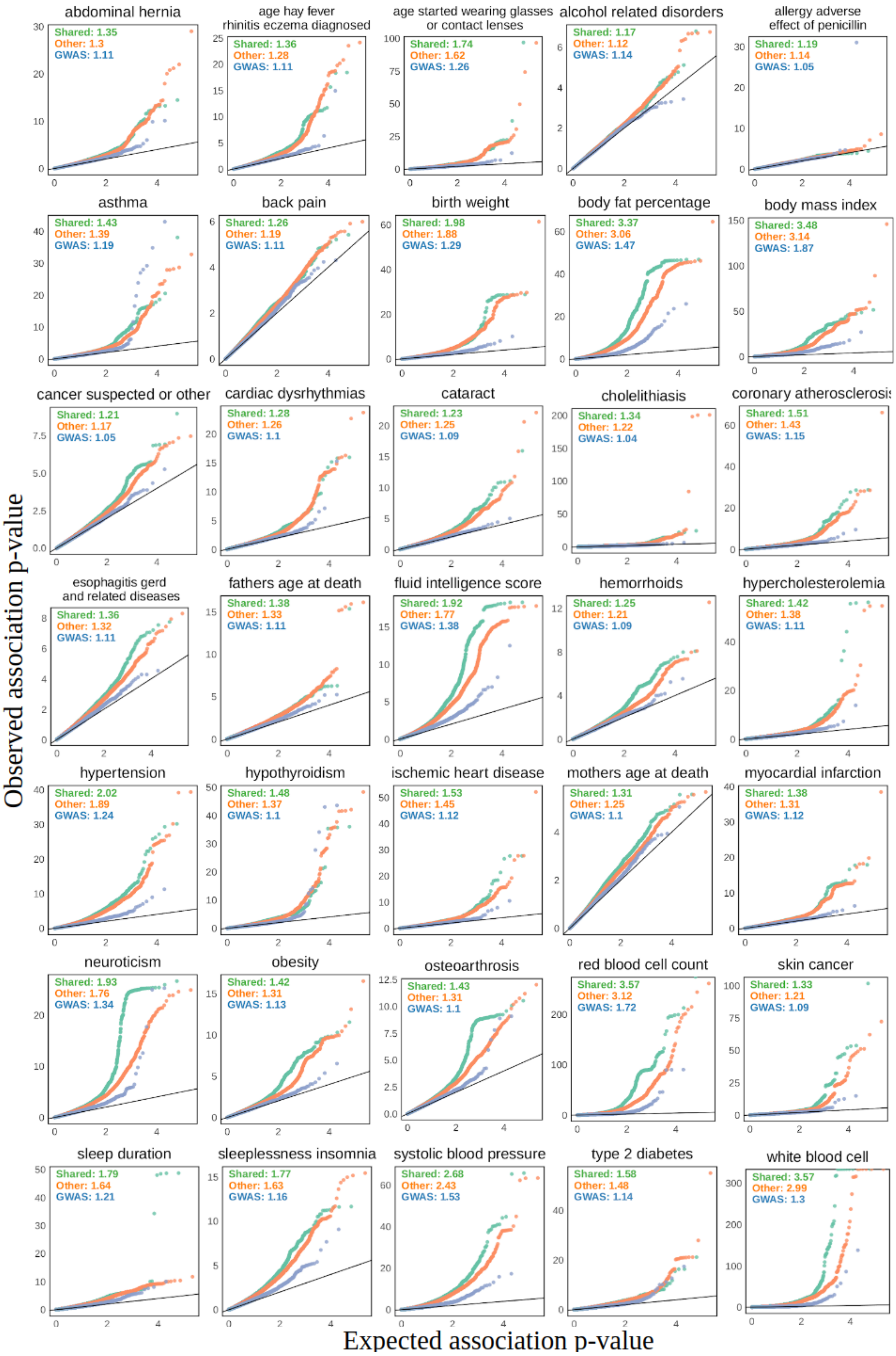


**Supplementary Fig. 25 Quantile-quantile (Q-Q) plots for shared lead eQTLs and other lead eQTLs from all GTEx tissues across 35 traits.** Shared (green) and other lead eQTLs (orange) were gathered from all 49

GTEx tissues. Values on the plot denote the genomic inflation factor (Methods). Note that the inflation is higher for shared lead eQTLs than other eQTLs across all but one (cataract) of the 35 traits. GWAS (blue) is a sample of 10000 variants (randomly and independently picked for each trait) shown for comparison purposes.

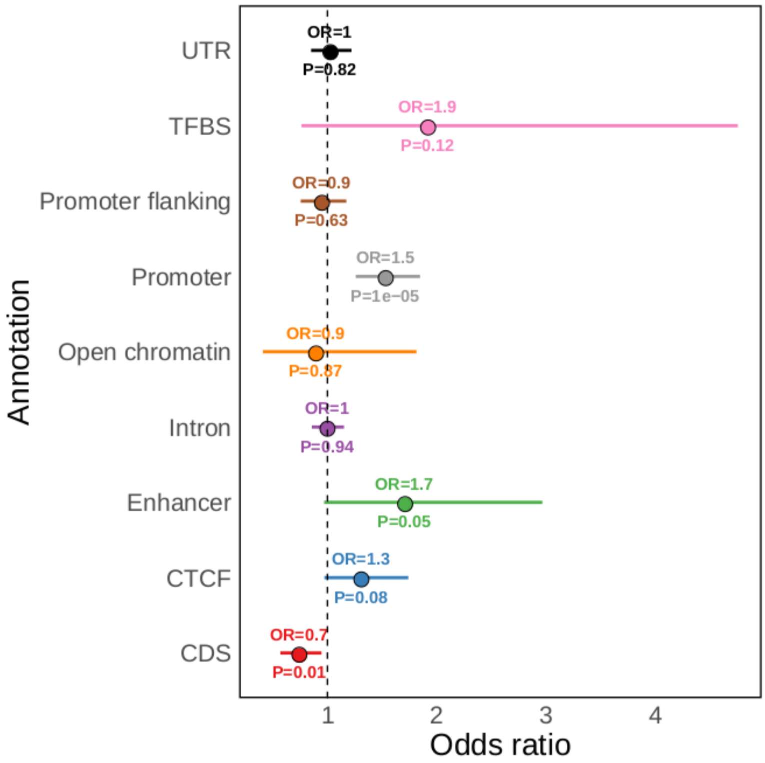


**Supplementary Fig. 26 Functional enrichment of shared and other pleiotropic variants.** Fisher's exact test odds ratio and p-value for the enrichment of pleiotropic shared lead eQTLs (N = 1274) in each functional annotation, compared to other pleiotropic lead eQTLs (N = 2647). Pleiotropic variants are defined as being associated ($P < 5e^{-8}$) with more than one of the 35 GWAS traits assessed, variants were gathered across 49 GTEx tissues. Error bars are 95% confidence intervals.

# Supplementary Tables

**Supplementary Table 1: Coefficients of logistic regression and two-way ANOVA F-values of the model including all molecular features tested.** All COPs were used for this (not only the training set). Note that the various features are not on the same scale.

| Metric | Estimate | Std.Error | z-value | Pr(>\|z\|) regression | F value (ANOVA) | Pr(>F) (ANOVA) |
|---|---|---|---|---|---|---|
| (Intercept) | 1.804439 | 0.092160 | 19.58 | <2E-16 | NA | NA |
| distance | 0.000001 | 0.000000 | 6.72 | 1.8E-11 | 0 | 1.0E+00 |
| totalCTCF | -0.005624 | 0.001204 | -4.67 | 3.0E-06 | 479.25 | <2E-16 |
| invertedCTCF | -0.021243 | 0.012248 | -1.73 | 8.3E-02 | 13.33 | 2.6E-04 |
| tssContact | -0.000048 | 0.000072 | -0.67 | 5.1E-01 | 0 | 9.9E-01 |
| totalEnhancers | -0.022587 | 0.004198 | -5.38 | 7.4E-08 | 419.04 | <2E-16 |
| sharedEnhancers | -0.032613 | 0.006321 | -5.16 | 2.5E-07 | 26.32 | 2.9E-07 |
| totalTFBS | -0.001119 | 0.000178 | -6.3 | 3.0E-10 | 254.7 | <2E-16 |
| sharedTF | 0.000685 | 0.003063 | 0.22 | 8.2E-01 | 0.14 | 7.1E-01 |
| diffExpr | -0.541101 | 0.037617 | -14.38 | <2E-16 | 970.97 | <2E-16 |
| diffCoef | -0.829129 | 0.053758 | -15.42 | <2E-16 | 437.22 | <2E-16 |
| LD_R2 | -0.330975 | 0.071803 | -4.61 | 4.0E-06 | 0.28 | 5.9E-01 |
| goSharing | 1.757654 | 0.122903 | 14.33 | <2E-16 | 187.38 | <2E-16 |
| eqtlSharing | 2.217305 | 0.055173 | 40.19 | <2E-16 | 2554.86 | <2E-16 |
| eGenes | -0.578565 | 0.033257 | -17.4 | <2E-16 | 371.97 | <2E-16 |

**Supplementary Table 2: Details of the 35 traits from Pan UK BioBank used in the study.**

| Description | Trait type | Phenocode | N_cases_EUR | N_controls_EUR |
|---|---|---|---|---|
| Abdominal hernia | phecode | 550 | 56668 | 363863 |
| Age hay fever, rhinitis or eczema diagnosed | continuous | 3761 | 83628 | NA |
| Age started wearing glasses or contact lenses | continuous | 2217 | 361940 | NA |
| Alcohol-related disorders | phecode | 317 | 16070 | 392046 |
| Allergy/adverse effect of penicillin | phecode | 960.2 | 20021 | 383239 |
| Asthma | phecode | 495 | 31169 | 379656 |
| Back pain | phecode | 760 | 17794 | 402737 |
| Birth weight | continuous | 20022 | 239716 | NA |
| Body fat percentage | continuous | 23099 | 412960 | NA |
| Body mass index (BMI) | continuous | 21001 | 419163 | NA |
| Cancer, suspected or other | phecode | 195 | 37387 | 367856 |
| Cardiac dysrhythmias | phecode | 427 | 31341 | 384657 |
| Cataract | phecode | 366 | 27820 | 392711 |
| Cholelithiasis | phecode | 574.1 | 17278 | 399542 |
| Coronary atherosclerosis | phecode | 411.4 | 23888 | 382052 |
| Esophagitis, GERD and related diseases | phecode | 530.1 | 40018 | 371349 |
| Father's age at death | continuous | 1807 | 310232 | NA |
| Fluid intelligence score | continuous | 20016 | 135088 | NA |

| | | | | |
|---|---|---|---|---|
| Hemorrhoids | phecode | 455 | 26726 | 376206 |
| Hypercholesterolemia | phecode | 272.11 | 40851 | 376397 |
| Hypertension | phecode | 401 | 94311 | 325488 |
| Hypothyroidism | phecode | 244 | 18404 | 399034 |
| Ischemic Heart Disease | phecode | 411 | 37672 | 382052 |
| Mother's age at death | continuous | 3526 | 249247 | NA |
| Myocardial infarction | phecode | 411.2 | 15065 | 382052 |
| Neuroticism score | continuous | 20127 | 341239 | NA |
| Obesity | phecode | 278.1 | 15917 | 404444 |
| Osteoarthrosis | phecode | 740 | 36073 | 384458 |
| Red blood cell (erythrocyte) count | continuous | 30010 | 408007 | NA |
| Skin cancer | phecode | 172 | 17691 | 402691 |
| Sleep duration | continuous | 1160 | 418009 | NA |
| Sleeplessness / insomnia | continuous | 1200 | 420013 | NA |
| Systolic blood pressure, automated reading | continuous | 4080 | 396663 | NA |
| Type 2 diabetes | phecode | 250.2 | 22768 | 396181 |
| White blood cell (leukocyte) count | continuous | 30000 | 408002 | NA |

# Supplementary Note 1: Analysis of negatively-correlated COPs

To compare the molecular feature signature between positively and negatively correlated COPs, we split these two categories of COPs and created a distance-matched set of non-COPs for each category. Overall, we found a similar molecular feature signature between positive and negative COPs (Supplementary Fig. 27 and 28). Main differences are *(i)* the AUC for ‘Expression’ (i.e. expression level difference and expression coefficient of variation difference) is lower for negative COPs, as expected for negative correlation; *(ii)* the AUC of LD is higher for negative COPs, indicating negative COPs are more genetically linked than expected; *(iii)* the AUC for eQTL sharing (regardless of effect sign) is high for negative COPs, however this could partially driven by an higher LD in negative COPs. Notably, the decrease of the regulatory complexity compared to non-COPs is still observed for negative COPs, in particular, a lower number of enhancers (Supplementary Fig. 28).

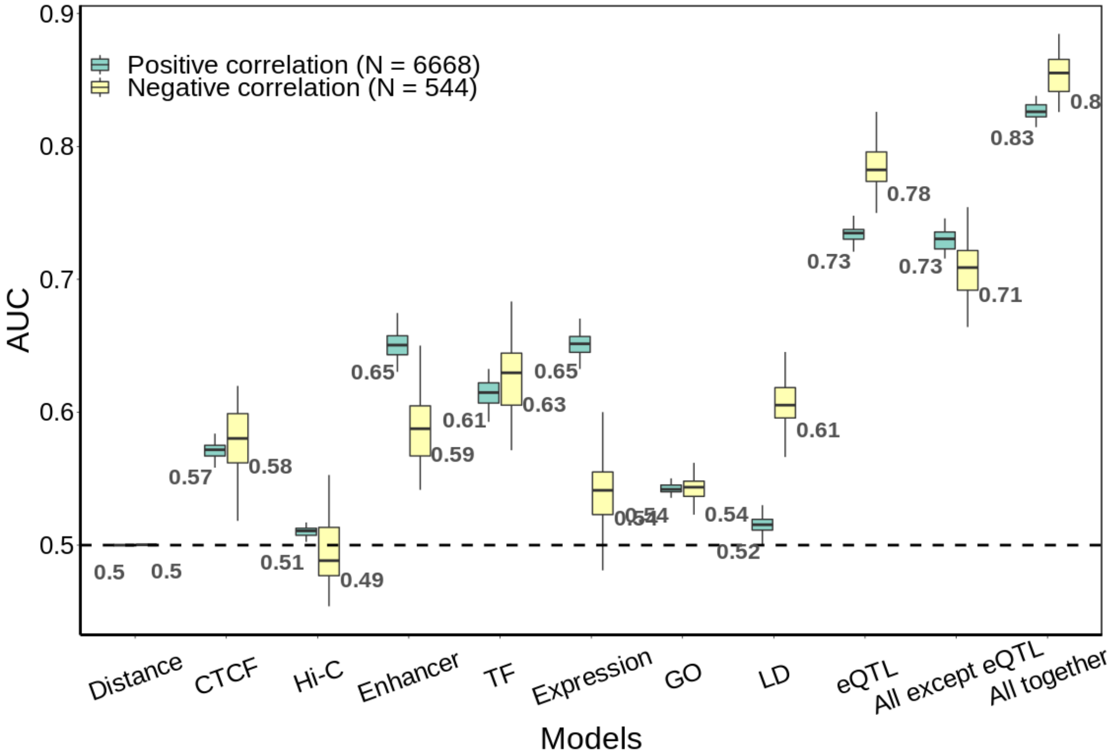


**Supplementary Fig. 27 Geuvadis LCL boxplots of the AUC values obtained for each molecular feature separated by positively and negatively correlated COPs.** Values below the boxplot represent the mean over the 50 randomisations. Note that positive correlation and negative correlation datasets were matched for distance distribution separately (i.e. non-COPs match appropriately each dataset of COPs). Note: for positive correlation eQTL sharing is only considered if the effect sign is matched, but for negative correlation consistency of the effect sign was not required. For each boxplot, the length of the box corresponds to the IQR with the centre line corresponding to the median, the upper and lower whiskers represent the largest or lowest value no further than 1.5 * IQR from the third and first quartile, respectively.

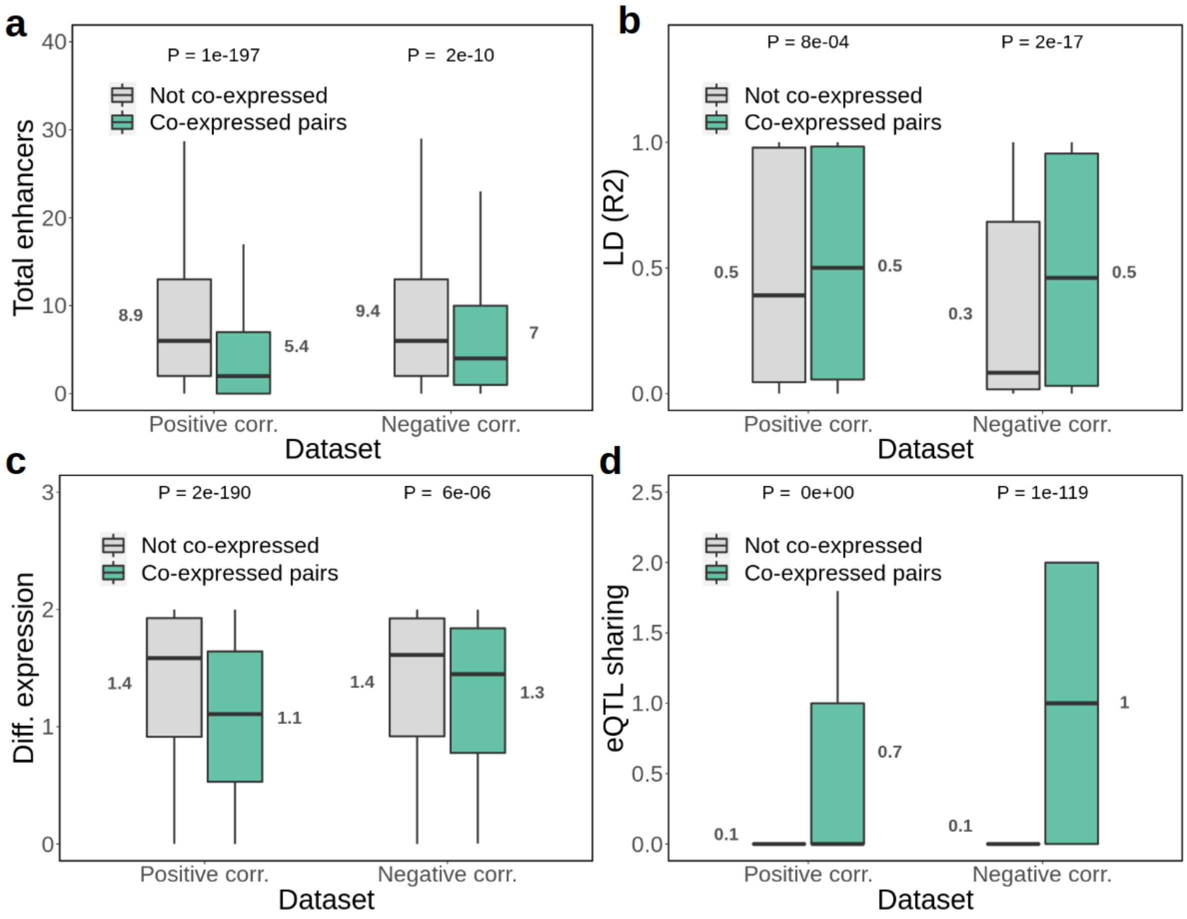


**Supplementary Fig. 28 Molecular feature boxplots comparing positively and negatively correlated COPs for (a) total enhancers, (b) linkage disequilibrium (LD) measured as $R^2$, (c) expression level difference and (d) eQTL sharing.** Positively correlated COPs N = 6668, negatively correlated COPs N = 544, for both COPs and distance-matched non-COPs in all plots. Values next to the boxplots represent the mean. P-values were obtained from two-tailed Wilcoxon signed-rank tests. Note: for positive correlation, eQTL sharing is only considered if the effect sign is matched, but for negative correlation the consistency of the effect sign was not required. For each boxplot, the length of the box corresponds to the IQR with the centre line corresponding to the median, the upper and lower whiskers represent the largest or lowest value no further than 1.5 * IQR from the third and first quartile, respectively.

Next, we analysed negatively correlated COPs in GTEx, where we find a total of 8,527 distinct negative COPs. This compares to 64,320 distinct positive COPs. Regarding tissue-specificity, positively correlated COPs show to be more widespread across tissues than negatively correlated COPs (Supplementary Fig. 29). On average, positively correlated COPs are present in 3.2 tissues, whereas negative COPs are present in only 1.5 tissues.

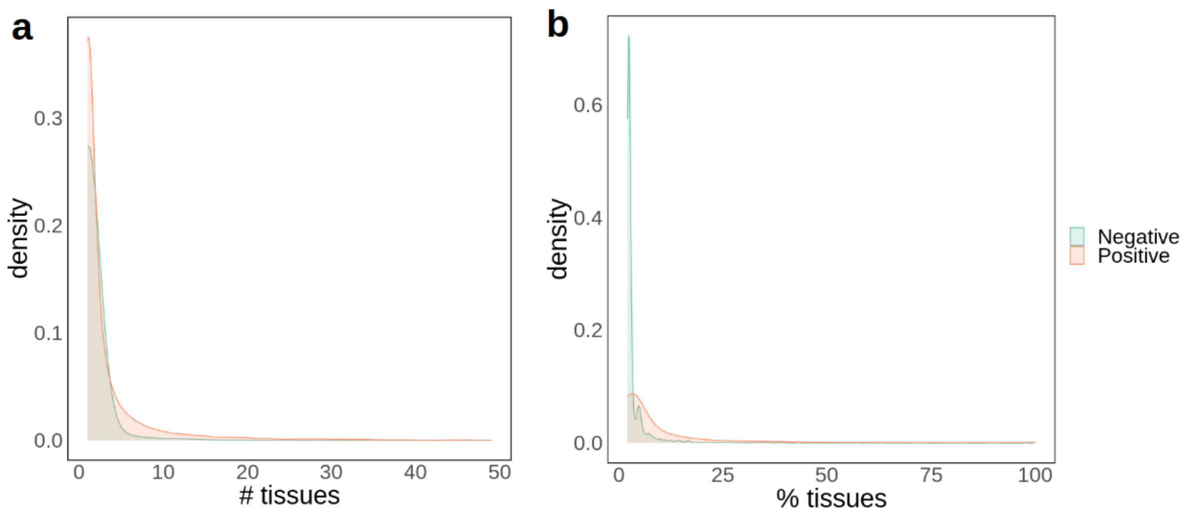


**Supplementary Fig. 29 COP tissue conservation comparing negatively and positively correlated COPs.** (a) distribution of the number of tissues where COP is present; (b) percentage of tissues where COP is found, out of all tissues where the gene pair was assessed. In this panel only gene pairs present in >5 tissues were considered, in order to exclude cases where present is 100% while in fact the gene pair was only assessed in a few tissues.

Finally, following up on the finding that negatively correlated COPs also display high eQTL sharing (albeit with opposite sign effect), we compared the functional enrichment of shared lead eQTLs between positively and negatively correlated COPs (Supplementary Fig. 30). The main difference between positive and negative eQTLs is a higher enrichment of negative eQTLs in the UTR region of genes (Supplementary Fig. 30). Otherwise, negative-related eQTLs display a similar enrichment against the expected background as positive-related eQTLs, such as a high enrichment in enhancer, protein binding and DNAse regions.

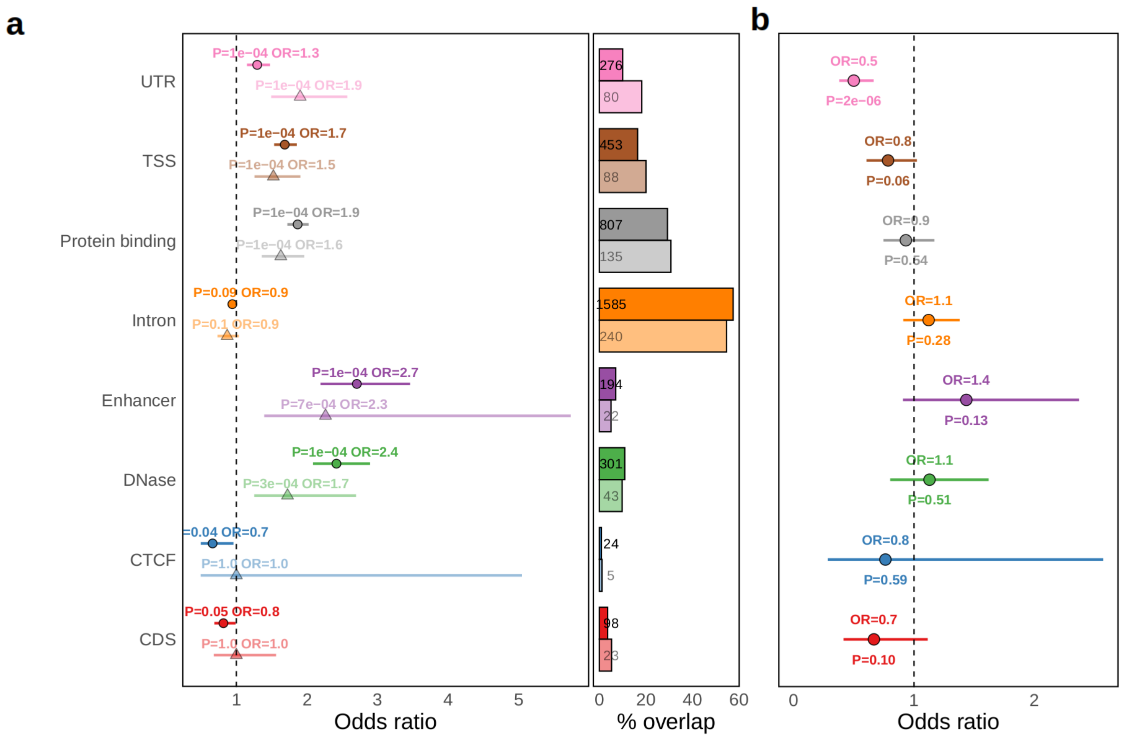


**Supplementary Fig. 30 Comparison of functional enrichment of shared eQTLs in positively and negatively correlated COPs.** (a) overlap enrichment of Geuvadis LCL lead shared eQTLs associated with positive COPs (solid color, round points) and lead shared eQTLs associated with negative COPs (pale color,

triangles) in Encode LCL functional annotations and Gencode gene body categories. Odd ratios are calculated based on the observed versus expected overlap (10000 QTLtools fenrich permutations; Methods) between eQTLs and each functional annotation, through two-sided Fisher's exact tests (no multiple test adjustment). Error bars are 95% confidence intervals. The right part of the plot denotes the percentage of overlap between eQTLs and each functional annotation; (b) Two-way Fisher's exact test odds ratio and p-value for the enrichment of positive-related eQTLs in each functional annotation, compared to negative-related eQTLs. Error bars are 95% confidence intervals.

Overall, we find negative COPs to be less numerous than positive COPs and display higher tissue-specificity. Yet, a similar molecular feature signature and the finding that shared eQTL in negative COPs fall in regulatory regions suggests this negative correlation, like positive correlation, may still be regulated by regulatory elements and genetic variation. Mechanisms such as enhancers or TFBS with repressing and activating activity for different genes could play a role in the regulation of negatively correlated COPs.

# Supplementary Note 2: Molecular feature comparison between cis-COPs and trans-COPs

To investigate how Geuvadis LCL cis-COP molecular features compare to those of trans-COPs, we defined trans-COPs as having correlation above 0.143 (Methods), and trans-non-COPs as gene pairs having low correlation value (i.e. <0.01). Then, for each cis-COP, a random trans-COPs with a similar correlation value (i.e. at most 5% difference) from the cis-COP correlation value was selected. This resulted in 6316 cis-COP/trans-COP matches (and 6316 corresponding cis-non-COPs and trans-non-COPs). Several molecular features such as enhancer sharing and counting CTCF sites between genes can only be performed for cis-COPs. We thus performed a molecular feature analysis for total TFBS, shared TFs, GO term sharing and difference in expression level/variation.

First we computed the AUCs for discriminating trans-COPs versus trans-non-COPs. We found the expression level difference/variation to have the highest AUC (0.73, Supplementary Fig. 31), a value higher than for cis-COPs (0.64). This difference is mostly driven by the fact that trans-non-COPs have more divergent expression than cis-non-COPs (Supplementary Fig. 32). In terms of the transcription factor features, these are less discrimitative of co-expression for trans-COPs (AUC 0.55) than for cis-COPs (AUC 0.61). However, we still found that trans-COPs display a lower amount of transcription factor presence around the TSS region compared to trans-non-COPs, indicating a similar evolutionary pressure as for local gene co-expression (Supplementary Fig. 32).

Next, we directly compared trans-COPs and cis-COPs. Here we find that the main discriminating feature between trans and cis COPs is the TF metrics (AUC = 0.75, Supplementary Fig. 31). In fact, while the number of total TFBS is very similar between cis-COPs and trans-COPs, cis-COPs tend to have higher transcription factor sharing (mean 23 for cis-COPs, 16.9 for trans-COPs). However, we observe even higher transcription factor sharing (mean 26.6, Supplementary Fig. 32) for cis-non-COPs, which indicates that the distribution of transcription factor binding sites around nearby gene pairs drives the distinction between trans-COPs and cis-COPs. Finally, GO term sharing shows similar levels across categories, indicating that functional similarity pressures are alike for cis-COPs and trans-COPs.

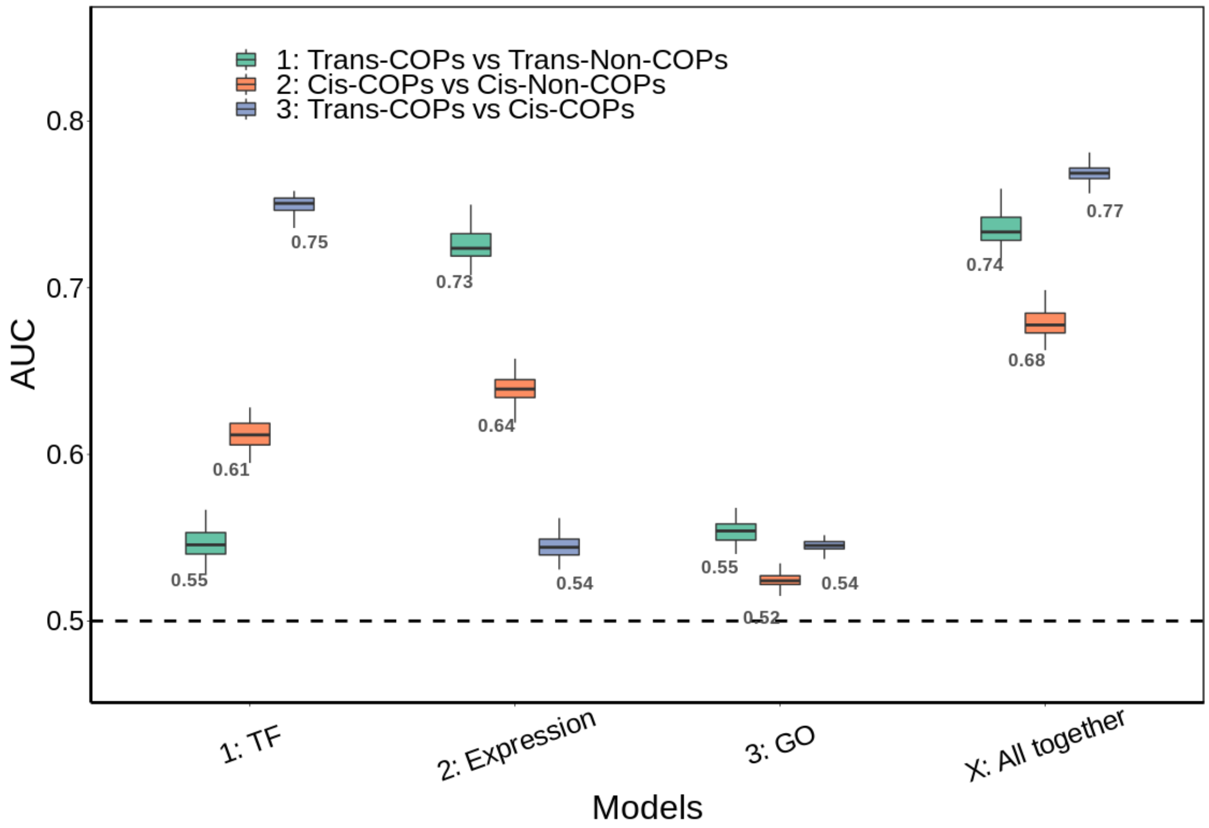


**Supplementary Fig. 31 Molecular features of 1) trans-COPs versus trans-non-COPs, 2) cis-COPs versus cis-non-COPs and 3) trans-COPs versus cis-COPs.** Same metrics and parameters as in the manuscript were used (80% train set, 20% test set). Boxplots are produced from 50 randomisations of the test/training set. N = 6316 for each category. For each boxplot, the length of the box corresponds to the IQR with the centre line corresponding to the median, the upper and lower whiskers represent the largest or lowest value no further than 1.5 * IQR from the third and first quartile, respectively.

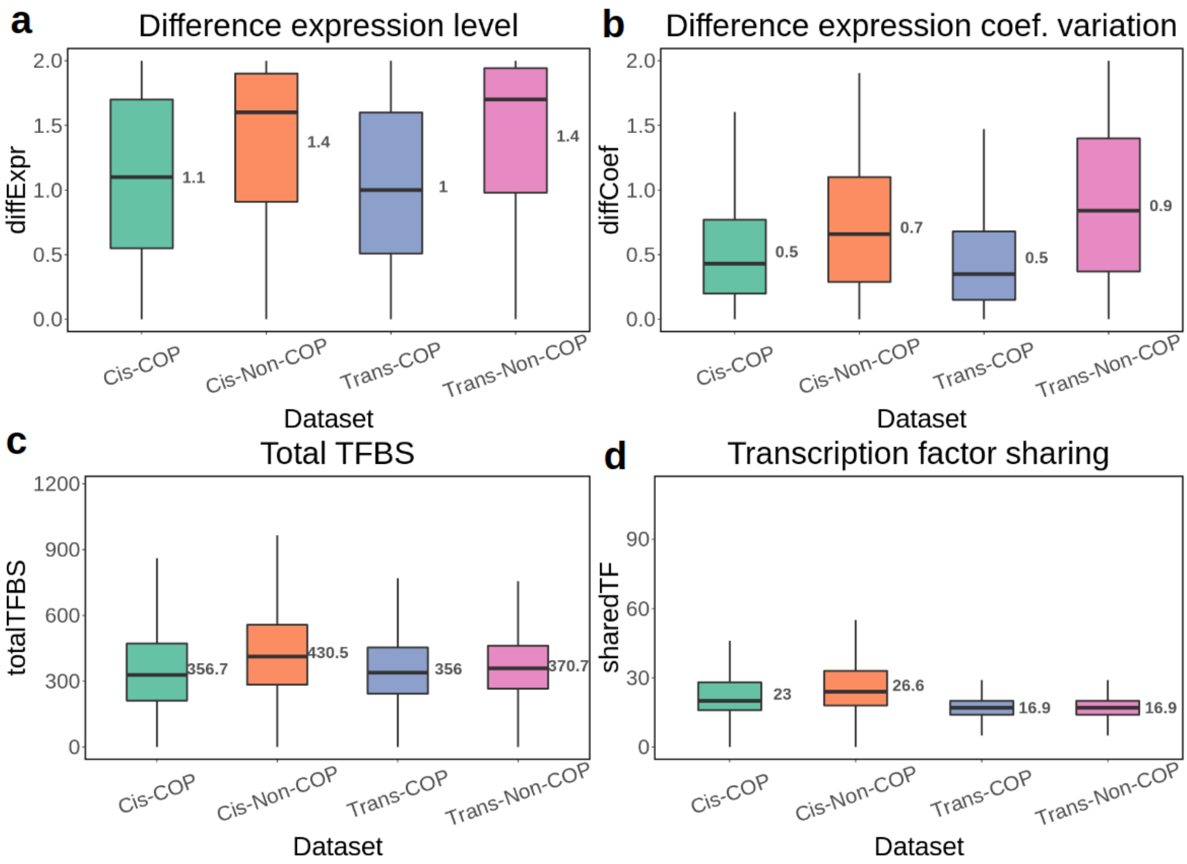


**Supplementary Fig. 32 Details of the molecular features across COP and non-COP datasets.** **a** expression level difference; **b** expression coefficient of variation difference; **c** total transcription factor binding sites (around 50Kb of TSS); **d** shared TFs, i.e. number of distinct transcription factor motifs shared between the gene pair. N = 6316 for each category across all plots. For each boxplot, the length of the box corresponds to the IQR with the centre line corresponding to the median, the upper and lower whiskers represent the largest or lowest value no further than 1.5 * IQR from the third and first quartile, respectively.
