# Supplementary material for: The molecular basis, genetic control and pleiotropic effects of local gene co-expression
Source: Nat Commun. 2021 Aug 10;12:4842. doi: 10.1038/s41467-021-25129-x (PMC8355184; doi:10.1038/s41467-021-25129-x)
Supplement: Supplementary file 3 — Description of Additional Supplementary Files [file 41467_2021_25129_MOESM3_ESM.pdf]

Description of Additional Supplementary Files

File Name: Supplementary Data 1
Description: List of eQTL-COP-tissue combinations that affect multiple traits generated in this study
