# Supplementary material for: The molecular basis, genetic control and pleiotropic effects of local gene co-expression
Source: Nat Commun. 2021 Aug 10;12:4842. doi: 10.1038/s41467-021-25129-x (PMC8355184; doi:10.1038/s41467-021-25129-x)
Supplement: Supplementary file 5 — Reporting Summary [file 41467_2021_25129_MOESM5_ESM.pdf]

nature research

Corresponding author(s): Olivier Delaneau

Last updated by author(s): Apr 9, 2021

Reporting Summary

Nature Research wishes to improve the reproducibility of the work that we publish. This form provides structure for consistency and transparency in reporting. For further information on Nature Research policies, see our Editorial Policies and the Editorial Policy Checklist.

Statistics

For all statistical analyses, confirm that the following items are present in the figure legend, table legend, main text, or Methods section.

n/a	Confirmed	
☐	☒	The exact sample size (n) for each experimental group/condition, given as a discrete number and unit of measurement
☒	☐	A statement on whether measurements were taken from distinct samples or whether the same sample was measured repeatedly
☐	☒	The statistical test(s) used AND whether they are one- or two-sided<br>*Only common tests should be described solely by name; describe more complex techniques in the Methods section.*
☐	☒	A description of all covariates tested
☐	☒	A description of any assumptions or corrections, such as tests of normality and adjustment for multiple comparisons
☐	☒	A full description of the statistical parameters including central tendency (e.g. means) or other basic estimates (e.g. regression coefficient) AND variation (e.g. standard deviation) or associated estimates of uncertainty (e.g. confidence intervals)
☐	☒	For null hypothesis testing, the test statistic (e.g. F, t, r) with confidence intervals, effect sizes, degrees of freedom and P value noted<br>*Give P values as exact values whenever suitable.*
☒	☐	For Bayesian analysis, information on the choice of priors and Markov chain Monte Carlo settings
☒	☐	For hierarchical and complex designs, identification of the appropriate level for tests and full reporting of outcomes
☐	☒	Estimates of effect sizes (e.g. Cohen's d, Pearson's r), indicating how they were calculated

Our web collection on statistics for biologists contains articles on many of the points above.

Software and code

Policy information about availability of computer code

Data collection	No software was used for the data collection of this study.
Data analysis	All code used in this study is publicly and readily available:<br>Custom scripts: Python v3.6.7 and R v3.4.4<br>VCF file manipulation bcftools v1.8, using htslib 1.8<br>BED file manipulation: bedtools v2.29.2<br>Network plotting: Cytoscape 3.8.0<br>Genomic coordinate liftover: USCS liftOver tool (3 Oct 2019, no version)<br>Covariate correction: QTLtools v1.1<br>eQTL and enrichment analysis: QTLtools v1.1<br>Colocalization: COLOC v3.2.1 (R package) on R 3.5.1<br><br>Custom code used for analysis is available under: https://github.com/diogomribeiro/LoCOP

For manuscripts utilizing custom algorithms or software that are central to the research but not yet described in published literature, software must be made available to editors and reviewers. We strongly encourage code deposition in a community repository (e.g. GitHub). See the Nature Research guidelines for submitting code & software for further information.

Data

Policy information about availability of data

All manuscripts must include a data availability statement. This statement should provide the following information, where applicable:

- Accession codes, unique identifiers, or web links for publicly available datasets
- A list of figures that have associated raw data
- A description of any restrictions on data availability

Data on co-expressed genes and shared eQTLs discovered here are available for consultation and download through the LoCOP database (http://glcoex.unil.ch/) developed here. The list of eQTL-COP-tissue combinations that affect multiple traits generated in this study are provided in Supplementary Data 1. Geuvadis RNA-seq and genotype data are available under accession numbers: EBI ArrayExpress (accession code E-GEUV-1) for RNA-seq data and 1000 Genomes (http://ftp.1000genomes.ebi.ac.uk/vol1/ftp/release/20130502/) for the genotype data. GTEx RNA-seq and genotype data are available from dbGaP (accession: phs000424.v8.p2).

Field-specific reporting

Please select the one below that is the best fit for your research. If you are not sure, read the appropriate sections before making your selection.

[x] Life sciences [ ] Behavioural & social sciences [ ] Ecological, evolutionary & environmental sciences

For a reference copy of the document with all sections, see nature.com/documents/nr-reporting-summary-flat.pdf

Life sciences study design

All studies must disclose on these points even when the disclosure is negative.

Sample size	Sample sizes were defined by availability of public datasets (not a choice in this study).<br>For the Geuvadis dataset in lymphoblastoid cell lines, 358 European samples with RNA-seq and genotype were available.<br>For the GTEx v8 dataset, a different sample size per tissue was available (ranging from 73 to 706)
Data exclusions	No samples were excluded. These had been quality controlled beforehand.
Replication	All the software and data used for this study are publicly available for the replication of the results.
Randomization	No samples were not split into experimental groups. Analysis considered covariates such as sex and subpopulation structure.
Blinding	Blinding is not relevant to this study since no group allocation occurs.

Reporting for specific materials, systems and methods

We require information from authors about some types of materials, experimental systems and methods used in many studies. Here, indicate whether each material, system or method listed is relevant to your study. If you are not sure if a list item applies to your research, read the appropriate section before selecting a response.

Materials & experimental systems

n/a	Involved in the study
[x]	[ ] Antibodies
[x]	[ ] Eukaryotic cell lines
[x]	[ ] Palaeontology and archaeology
[x]	[ ] Animals and other organisms
[x]	[ ] Human research participants
[x]	[ ] Clinical data
[x]	[ ] Dual use research of concern

Methods

n/a	Involved in the study
[x]	[ ] ChIP-seq
[x]	[ ] Flow cytometry
[x]	[ ] MRI-based neuroimaging
